# Natural Sequence Variations and Combinations of *GNP1* and *NAL1* Determine the Grain Number per Panicle in Rice

**DOI:** 10.1186/s12284-020-00374-8

**Published:** 2020-02-28

**Authors:** Yun Wang, Laiyuan Zhai, Kai Chen, Congcong Shen, Yuntao Liang, Chunchao Wang, Xiuqin Zhao, Shu Wang, Jianlong Xu

**Affiliations:** 1 Rice Research Institute, Shenyang Agricultural University, Shenyang, 110866 Liaoning China; 2 Institute of Crop Sciences/National Key Facility for Crop Gene Resources and Genetic Improvement, Chinese Academy of Agricultural Sciences, Beijing, 100081 China; 3 Shenzhen Branch, Guangdong Laboratory for Lingnan Modern Agriculture, Agricultural Genomics Institute at Shenzhen, Chinese Academy of Agricultural Sciences, Shenzhen, 518120 Guangdong China; 4 Rice Research Institute, Guangxi Academy of Agricultural Sciences, Nanning, 530007 Guangxi China

**Keywords:** *NAL1*, *GNP1*, Pyramiding, Nucleotide diversity, Phenotypic variation

## Abstract

**Background:**

The grain number per panicle (GNP), which is one of three grain yield components, is an important trait for the genetic improvement of rice. Although the *NAL1* and *GNP1* genes regulating the rice GNP and grain yield have been cloned, their allelic diversity, functional differences in rice germplasms, and effects of their combination on GNP and grain yield remain unclear.

**Results:**

Based on DNA sequences of these two genes in 198 cultivated rice (*Oryza sativa*) and 8–10 wild rice (*Oryza rufipogon*) germplasms, 16 and 14 haplotypes were identified for *NAL1* and *GNP1*, respectively. The *NAL1* gene had the strongest effects on GNP in *indica* (*xian*) and *japonica* (*geng*) subpopulations. In contrast, *GNP1* had no significant effects in the *geng* subpopulation and was rare in the *xian* background, in which the superior *GNP1* allele (*GNP1*–6) was detected in only 4.0% of the 198 germplasms. Compared with the transgenic lines with *GNP1* or *NAL1*, the transgenic lines with both genes had a higher GNP (15.5%–25.4% and 11.6%–15.9% higher, respectively) and grain yield (5.7%–9.0% and 8.3%–12.3% higher, respectively) across 3 years. The two genes combined in the introgression lines in Lemont background resulted in especially favorable effects on the GNP.

**Conclusions:**

Our results indicated that the *GNP1* and *NAL1* exhibited obvious differentiation and their combinations can significantly increase the grain yield in *geng* rice cultivars. These observations provide insights into the molecular basis of the GNP and may be useful for rice breeding of high yield potential by pyramiding *GNP1* and *NAL1*.

## Background

Rice (*Oryza sativa* L.) is one of the most important staple food crops worldwide. Therefore, research aimed at increasing rice yield is critical for ensuring food production and satisfying the demands of a rapidly growing global population. Grain number per panicle (GNP), seed setting rate, effective panicle number per plant (EPN) and grain weight are the most important components of rice grain yield. Moreover, the GNP is also an important agronomic characteristic related to the ideal plant architecture (Virk et al. 2004). Increasing the rice grain yield by selective breeding involving grain yield components and the ideal plant architecture has been the key focus of rice breeding programs (Zhang 2007).

Map-based cloning methods have recently been applied to isolate several quantitative trait loci (QTLs) affecting yield-related traits, including *Gn1a* (Ashikari et al. 2005), *DEP1* (Huang et al. 2009), *OsSPL14* (Jiao et al. 2010), *NAL1* (*SPIKE*, *GPS*, *LSCHL4*, and *SS1*) (Fujita et al. 2013; Takai et al. 2013; Zhang et al. 2014; Xu et al. 2015), and *GNP1* (Wu et al. 2016). These genes control plant architecture and panicle type, ultimately influencing rice grain yield mainly through pleiotropic effects. *Narrow leaf 1* (*NAL1*), which affects leaf morphogenesis by regulating polar auxin transport, was first cloned from a loss-of-function narrow-leaf rice mutant by Qi et al. (2008). Previous studies revealed that *NAL1* is associated with pleiotropic effects that regulate the development of multiple traits related to the source (e.g., leaf width, leaf chlorophyll content, and photosynthetic efficiency), the sink (e.g., GNP), and the EPN in diverse genetic backgrounds (Fujita et al. 2013; Takai et al. 2013; Zhang et al., 2014; Xu et al. 2015; Yano et al. 2016). Another major QTL, *GNP1*, encodes the rice gibberellin biosynthesis gene *GA20ox1*. Increases in the GNP and rice grain yield involve enhanced cytokinin activity in panicle meristems. Additionally, rice plant height (PH) is regulated by *OsGA20ox1* (Oikawa et al. 2004). In our previous studies, we found that introgression of the superior *GNP1* allele from Teqing into Lemont lead to significantly increase in flag leaf length and area (Unpublished). So, *GNP1* also has pleiotropic effects on the development of multiple traits related to the source (e.g. leaf size) and the sink (e.g., GNP). The superior *NAL1* allele from the low-yielding Lemont variety can further increase 3.2%–3.8% grain yield of high-yielding Teqing variety in different environments (Xu et al. 2015), while the superior *GNP1* allele from the high-yielding Teqing variety can increase 5.7%–9.6% grain yield of low-yielding Lemont variety in different environments (Wu et al. 2016). These results suggested that superior *NAL1* and *GNP1* alleles can potentially be used in high yield rice breeding. However, the allelic diversity and functional differences of *GNP1* and *NAL1* in rice germplasms remain unclear. Moreover, their relative importance and pyramiding effect on grain yield have not been characterized.

In this study, we re-sequenced *GNP1* and *NAL1* from 198 cultivated rice (*Oryza sativa*) and 8–10 wild rice (*Oryza rufipogon*) germplasms. The allelic frequencies and the associated differences in the GNP were compared between the *xian* and *geng* subpopulations. Furthermore, the potential utility of combining *NAL1* and *GNP1* to increase the rice grain yield was investigated by comparing the lines with either *NAL1* or *GNP1* with the lines carrying both *NAL1* and *GNP1*. The results presented herein may help to clarify the comprehensive role of these two genes alone and in combination on the GNP and the potential rice grain yield. This study may also be useful for developing an efficient way to increase the GNP, thereby increasing the overall rice grain yield.

## Results

### Phenotypic and Genetic Variations in the Rice Germplasms

The 198 cultivated rice germplasms varied considerably regarding the GNP, flag leaf width (FLW), and PH (Additional file 1: Figure S1). The scree plot indicated that two principal components should be retained for the principal component analysis (Additional file 2: Figure S2a). The principal component score plot revealed the distribution of each accession in the rice diversity panel (Additional file 2: Figure S2b). Additionally, the highest log-likelihood scores of the population structure were obtained when the number of populations was set at 2 (K = 2; Additional file 2: Figure S2c), indicating the existence of two distinct subpopulations in the current panel (Additional file 2: Figure S2d, Additional file 3: Table S1). These findings along with the available accession information resulted in the identification of 146 *xian* and 43 *geng* varieties as well as three *Aus* and six admixed varieties. There were significant differences (*P* < 0.05) in the GNP and FLW, but not in the PH, between the *xian* and *geng* subpopulations over 3 years (Additional file 1: Figure S1).

### Nucleotide Variations in *GNP1* and *NAL1*

The 198 *O. sativa* germplasms were examined regarding the diversity in their *GNP1* and *NAL1* sequences. The 2.48 single nucleotide polymorphisms (SNPs) per kilobase (π = 2.48 × 10^− 3^) of the *GNP1* locus was slightly fewer than 2.64 SNPs per kilobase (π = 2.64 × 10^− 3^) in *O. rufipogon* (Additional file 4: Table S2). A selection signal was not detected in the neutrality tests of the whole, *xian*, and *geng* populations (Additional file 4: Table S2). A sliding-window analysis indicated the π value was highest in the promoter region (Additional file 5: Figure S3a).

The nucleotide diversity at the *NAL1* locus in the *xian* and *geng* germplasms and in *O. rufipogon* was 1.14 × 10^− 3^, 0.88 × 10^− 3^, and 1.0 × 10^− 3^, respectively (Additional file 4: Table S2). The π value in *O. sativa* (π = 1.65 × 10^− 3^) was nearly 2-fold higher than that in *O. rufipogon*. A neutrality test revealed a lack of selection signals in both subpopulations (Additional file 4: Table S2). The results of a sliding-window analysis revealed that in the *geng* subpopulation, the π value was highest in the third exon and in the 3′ untranslated region (UTR) (Additional file 5: Figure S3b).

### Analysis of the *GNP1* Haplotype

**Fig. 1:**
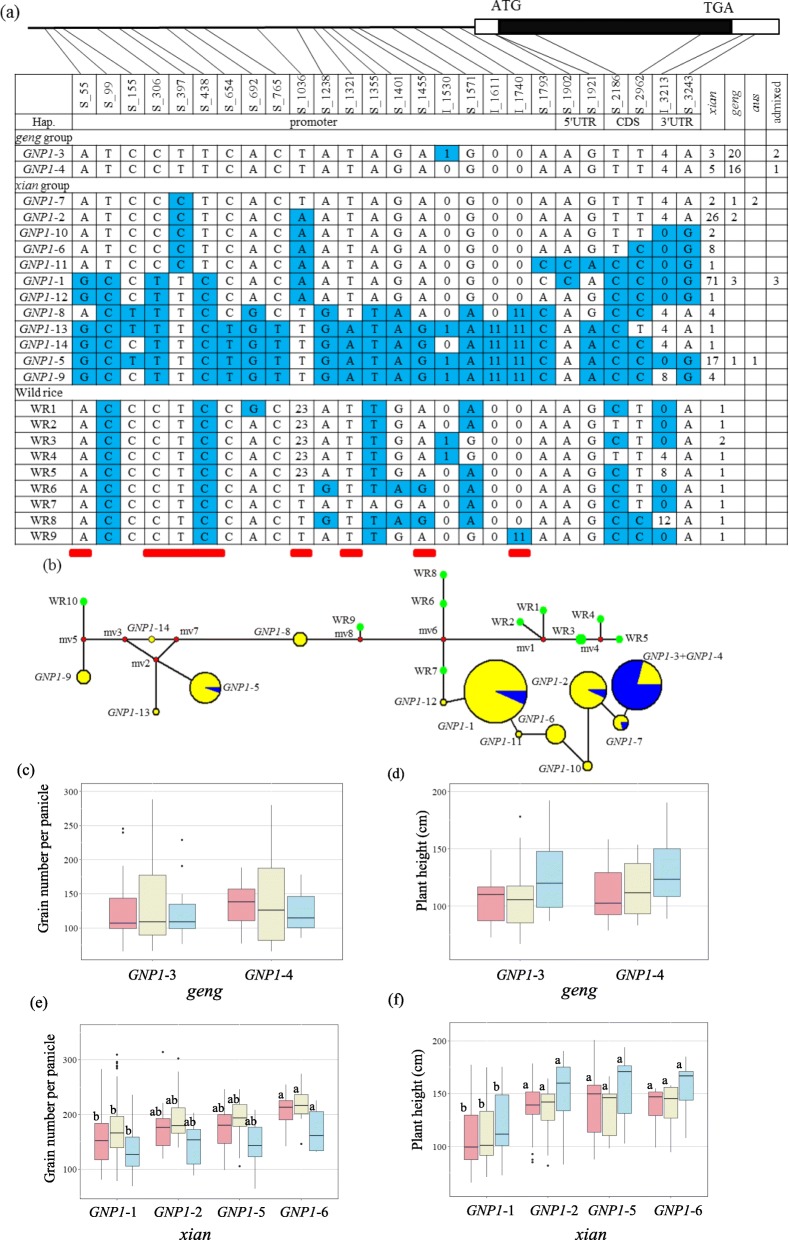
*GNP1* haplotype analysis. **a** The gene structure of *GNP1*. The coding region is indicated as black box, 3′ UTR and 5′ UTR are indicated as white boxes, and single nucleotide polymorphism (SNP) positions are connected to the haplotype table by lines (SNP frequency > 5%). Six potential intervals of promoters, marked with red stick, are found in the promoter region by promoter prediction software. Fourteen haplotypes, classified by SNP and insertion/deletion (InDel) variations, are detected in the collection (indicated as either blue or white). Numbers show the length of the InDel in bp. The number and subpopulation identity of varieties carrying each haplotype are indicated in columns to the right. The ten wild rice (*O.rufipogon*) varieties are indicated by WR1–9. **b** The phylogenetic network of *GNP1*. The phylogenetic network of haplotypes in *Oryza sativa* and *O. rufipogon* are constructed for *GNP1*. Each circle represents an allele. The size of the circles indicates the frequency of each allele. The green, yellow and blue circles indicate the allelic distribution in the wild rice, *xian* and *geng* species, respectively. The red median vectors (mv) represent a hypothesized sequence that is required to connect the existing sequences within the network with maximum parsimony. The solid lines represent one mutation step that interconnects adjacent alleles. **c**–**d** Grain number per panicle (**c**), plant height (**d**) are compared between the *GNP1*–3 and *GNP1*–4 haplotypes in *geng* rice (*Oryza sativa*). **e**–**f** Grain number per panicle (**e**), plant height (**f**) are compared among the *GNP1*–1,*GNP1*–2,*GNP1*–5 and *GNP1*–6 haplotypes in *xian* rice (*Oryza sativa*). Letters on histograms (a, and b) are ranked by Duncan’s test at *P* < 0.05. Pink, light yellow and light blue colors indicate in 2014, 2015 and 2016, respectively

The complete *GNP1* genomic sequence (3.7 kb) in the 198 analyzed accessions was re-sequenced. A total of 22 SNPs and four insertions/deletions (InDels) with a minimum allele frequency > 5% were considered for a haplotype analysis. Of these polymorphisms, 19 SNPs and three InDels were located in the promoter and 5′ UTR, including two 11-bp InDels in the promoter region. Additionally, one synonymous SNP and one nonsynonymous SNP leading to the replacement of an alanine by a valine were present in the coding region, with the remaining SNP and InDel located in the 3′ UTR (Fig. 1a). The amino acid variations detected in this study were the same as those identified in one of our previous studies (Wu et al. 2016).

Fourteen haplotypes (*GNP1*–1 to *GNP1*–14) were constructed based on the SNPs and InDels. A phylogenetic network for *GNP1* alleles was constructed based on the 22 SNPs and four InDels in *O. sativa* and *O. rufipogon* (Fig. 1b). Accordingly, we determined that the most prevalent haplotypes were *GNP1*–1, *GNP1*–2, *GNP1*–3, *GNP1*–4, and *GNP1*–5, which were detected in 77, 28, 25, 22, and 19 varieties, respectively (Fig. 1a). The next most prevalent haplotype was *GNP1*–6, which occurred in eight varieties. The other haplotypes were relatively rare (i.e., only in one to five varieties). Haplotypes *GNP1*–3 (represented by Lemont) and *GNP1*–4 were mainly present in accessions belonging to the *geng* subpopulation (i.e., 76.6%), whereas the other four haplotypes (*GNP1*–1, *GNP1*–2, *GNP1*–5, and *GNP1*–6) were predominantly detected in *xian* varieties (i.e., 92.4%) (Fig. 1b). Moreover, there was relatively little diversity in the *GNP1* sequence within the *geng* subpopulation, with only two haplotypes (*GNP1*–3 and *GNP1*–4) defined by one InDel at position I_1530 in the promoter region (Fig. 1a). In contrast, several *GNP1* haplotypes were identified in the *xian* subpopulation, in which haplotype *GNP1*–9 had the most variability in the SNP and InDels (92.3% polymorphic sites) as well as a large genetic distance from *GNP1–2* (Fig. 1b). Additionally, the *GNP1* allele in the *GNP1*–9 haplotype (present in four *xian* accessions) is identical to that in Teqing with the superior *GNP1* haplotype described by Wu et al. (2016). The differences in the GNP and PH were examined among varieties carrying haplotypes *GNP1*–1 to *GNP1*–6 because of the high frequency of these haplotypes in the rice germplasms. No differences in the GNP and PH were observed between the germplasms with the two major *geng* haplotypes (*GNP1*–3 and *GNP1*–4) in the *geng* subpopulation (Fig. 1c, d). However, in the *xian* subpopulation, the germplasms with the *GNP1*–6 haplotype had a higher GNP than those with *GNP1*–1. Additionally, the PH was lower for the germplasms with *GNP1*–1 than for the germplasms with the other three major *xian* haplotypes (*GNP1*–2, *GNP1*–5, and *GNP1*–6) (Fig. 1e, f). Thus, the germplasms carrying the *GNP1*–6 haplotype had a higher GNP and PH than those with *GNP1*–1.

In 198 panels, two protein types (*GNP1*-P1 and *GNP1*-P2) were identified based on one nonsynonymous SNP (Additional file 6: Figure S4a). There was no difference in GNP between the two protein types in *xian* subpopulation in 3 years except in 2014 (Additional file 6: Figure S4 b). Accessions carrying *GNP1*-P2 exhibited significantly higher PH than those carrying *GNP1*-P1 in *xian* subpopulation (Additional file 6: Figure S4 c).

To determine whether the SNPs or InDels in the promoter affected the *GNP1* locus at the transcriptional level, we compared the relative expressions of *GNP1*–6 and *GNP1*–1. In young panicles, *GNP1*–6 was more highly expressed than *GNP1*–1 (Additional file 7: Figure S5a).

### Analysis of the *NAL1* Haplotype

**Fig. 2:**
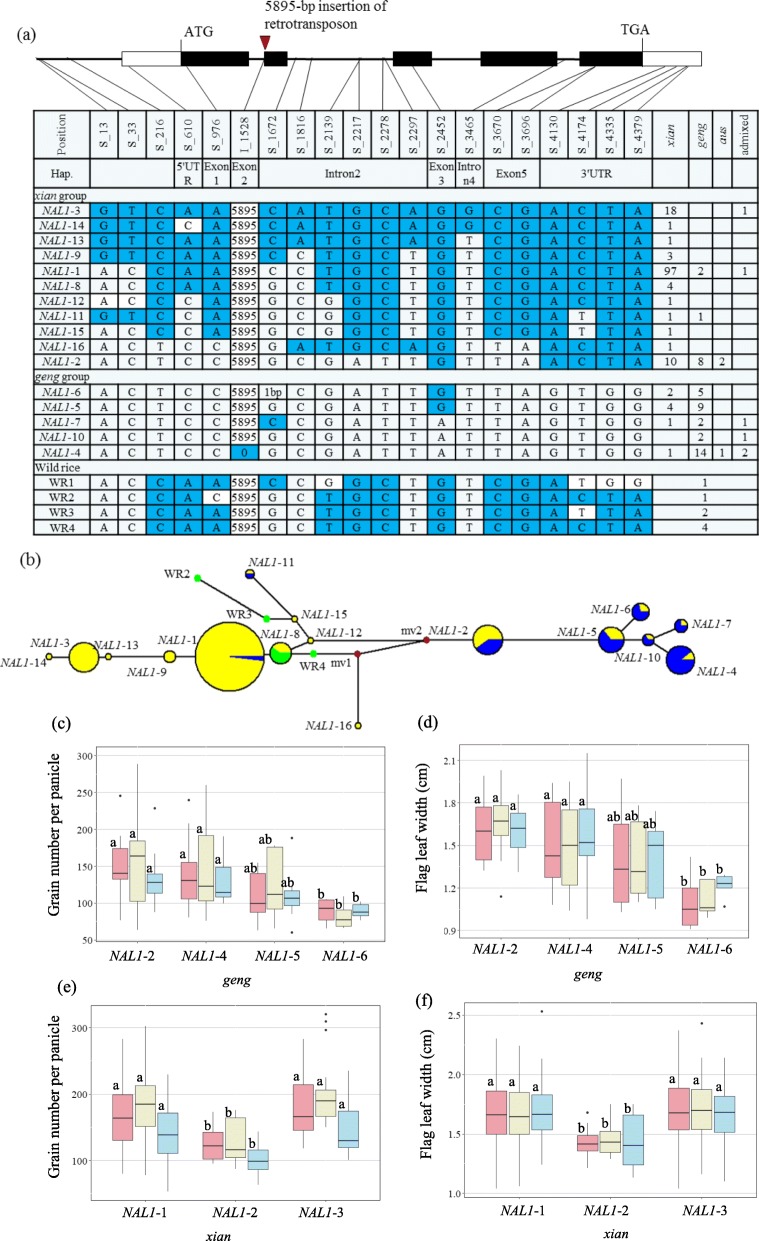
*NAL1* haplotype analysis. **a** The gene structure of *NAL1*. The five exons are indicated as black boxes, 3′ UTR and 5′ UTR are indicated as white boxes, and single nucleotide polymorphism (SNP) positions are connected to the haplotype table by lines (SNP frequency > 5%). Sixteen haplotypes, classified by SNP and insertion/deletion (InDel) variations, are detected in the collection (indicated as either blue or white). Numbers show the length of the InDel in bp. The number and subpopulation identity of varieties carrying each haplotype are indicated in columns to the right. The eight wild rice varieties of *O.rufipogon* are indicated by WR1–4. **b** The phylogenetic network of *NAL1*. The phylogenetic network of haplotypes in *Oryza sativa* and *O. rufipogon* are constructed for *NAL1*. **c**–**d** Grain number per panicle (**c**), flag leaf width (**d**) are compared among the *NAL1*–2, *NAL1*–4, *NAL1*–5 and *NAL1*–6 haplotypes in *geng* rice (*Oryza sativa*). **e**–**f** Grain number per panicle (**e**), flag leaf width (**f**) are compared among the *NAL1*–1, *NAL1*–2 and *NAL1*–3 haplotypes in *xian* rice (*Oryza sativa*). Letters on histograms (a, and b) are ranked by Duncan’s test at *P* < 0.05.Light pink, light yellow and light blue colors indicate in 2014, 2015 and 2016, respectively

We detected 19 SNPs and one InDel in the 4.4-kb *NAL1* genomic region. Four SNPs were located in the promoter. One synonymous SNP was detected in the first exon, whereas one nonsynonymous SNP in the third exon resulted in the replacement of an arginine by a histidine. Two nonsynonymous SNPs were located in the fifth exon. Of the remaining 11 SNPs, seven and four were located in the intron and the 3′ UTR, respectively. A 5895-bp retrotransposon insertion was identified in the junction between the first intron and the second exon of *NAL1* (Fig. 2a). However, the 10-amino acid deletion encoded in the fourth exon that was previously reported in the mutant Taichung 65 (Takai et al. 2013) was not detected in any of the 198 cultivated and eight wild rice germplasms, implying that this amino acid deletion is a rare mutation.

Sixteen haplotypes (*NAL1*–1 to *NAL1*–16) were identified (Fig. 2a). The *NAL1* phylogenetic network revealed the most prevalent haplotypes were *NAL1*–1 to *NAL1*–5, which were present in 100, 20, 19, 18, and 13 germplasms, respectively (Fig. 2b). The next most prevalent haplotype was *NAL1*–6, which was detected in seven germplasms. The other haplotypes were present in fewer than five varieties each. The *NAL1*–1 and *NAL1*–3 haplotypes were prevalent in the *xian* subpopulation (i.e., 97% and 94.7%, respectively), whereas the other three haplotypes (*NAL1*–4, *NAL1*–6, and *NAL1*–5) were mainly present in the *geng* germplasms (i.e., 77.8%, 71.4%, and 69.2%, respectively) (Fig. 2b). One admixed pattern was identified for *NAL1*–2, which was included in 10 (50%) *geng*, 8 (40%) *xian*, and 2 (10%) *Aus* accessions.

In the *geng* subpopulation, the germplasms with the *NAL1*–2 and *NAL1*–4 haplotypes had a higher GNP and a greater FLW than the germplasms with the *NAL1*–6 haplotype (Fig. 2c, d). In the *xian* subpopulation, the germplasms containing the *NAL1*–1 and *NAL1*–3 haplotypes had a considerably higher GNP and greater FLW than the germplasms with the *NAL1*–2 haplotype (Fig. 2e, f).

In 198 panels, three protein types (*NAL1*-P1, *NAL1*-P2 and *NAL1*-P3) were observed based on three nonsynonymous SNPs (Additional file 8: Figure S6 a). Accessions carrying *NAL1*-P1 had significantly more GNP and larger FLW than those carrying *NAL1*-P2 in *xian* subpopulation (Additional file 8: Figure S6 b–c). Accessions carrying *NAL1*-P3 possessed significantly more GNP than those with *NAL1*-P2 in *geng* subpopulation in 2014 and 2016 (Additional file 8: Figure S6 d). And varieties with *NAL1*-P3 showed significantly enlarged FLW compared with those with *NAL1*-P2 in *geng* subpopulation (Additional file 8: Figure S6 e).

The real-time PCR results demonstrated that *NAL1*–2 was more highly expressed in young panicle than *NAL1*–4 or *NAL1*–6 in the *geng* germplasms (Additional file 7: Figure S5b). However, there were no expression-level differences in the rice young panicle among the *NAL1*–1, *NAL1*–2, and *NAL1*–3 haplotypes in the *xian* germplasms (Additional file 7: Figure S5c).

### Association Analysis of SNPs and Traits

An association analysis of *GNP1* identified a SNP (S_397) located at 1662 bp upstream of the ATG (chromosome 3: 36,149,264) that was significantly associated with the GNP and PH (Table 1).

**Table 1:** Results of MLM association of SNPs extracted from the two genes (*NAL1* and *GNP1*) with traits in 198 accessions

Gene	Trait	GNP		FLW		PH	
	Site	*P*	*R*^*2*^ (%)	*P*	*R*^*2*^ (%)	*P*	*R*^*2*^ (%)
*NAL1*	S_1672	0.0019	6.70	–	–	–	–
	S_2452	0.0305	2.47	0.0071	4.21	–	–
*GNP1*	S_397	0.0125	3.37	–	–	0.0073	4.18

*R*^*2*^ (%) Phenotypic variance explained, *GNP* grain number per panicle, *FLW* flag leaf width, *PH* plant height

For *NAL1*, a SNP (S_2452) located in the third exon (chromosome 4: 31,212,801) leading to the replacement of an arginine by a histidine was significantly associated with the GNP and FLW (Table 1). Another SNP (S_1672) located in the second intron was significantly associated with the GNP (Table 1). An allele with a 1-bp deletion was detected only in haplotype *NAL1*–6 and was the only variation between haplotypes *NAL1*–6 and *NAL1*–5 (Fig. 2a).

### Effects of the Combination of Two Genes on the Grain Number per Panicle and the Grain Yield per Plant

On the basis of different major haplotypes for a single gene in 198 germplasms, the following four two-gene combinations were included in a comparative analysis of the *xian* subpopulation: *GNP1*–6/*NAL1*–1, *GNP1*–1/*NAL1*–1, *GNP1*–1/*NAL1*–2, and *GNP1*–1/*NAL1*–3 (Table 2). Six germplasms carrying *GNP1*–6/*NAL1*–1 had a higher GNP than the germplasms with the other three gene combinations, with average values of 201.7, 220.5, and 176.9 in 2014, 2015, and 2016, respectively (Table 2). Of the 71 *xian* germplasms with the *GNP1*–1 allele, 49, 6, and 6 had *NAL1*–1, *NAL1*–2, and *NAL1*–3, respectively. Six germplasms with the *GNP1*–1/*NAL1*–3 combination had a significantly higher GNP than the germplasms with *GNP1*–1/*NAL1*–2 (Table 2). However, the GNP did not significantly differ between the germplasms with *GNP1*–1/*NAL1*–1 or *GNP1*–1/*NAL1*–2 (Table 2), despite the observation that the germplasms with the *NAL1*–1 haplotype had a significantly higher GNP than the germplasms with *NAL1*–2 regardless of the genetic background (Fig. 2e). These observations implied that the *GNP1*–1 haplotype mainly affects the GNP.

**Table 2:** Comparison of grain number among genotype combinations at *NAL1* and *GNP1* in *xian* subpopulation

Haplotype	N	GNP		
		2014	2015	2016
*GNP1*–1/*NAL1*–3	6	197.9 ± 63.8a	212.3 ± 71.6a	161.3 ± 45.7a
*GNP1*–1/*NAL1*–1	49	158.4 ± 49.8ab	169.1 ± 52.3ab	130.7 ± 38.1ab
*GNP1*–1/*NAL1*–2	6	122.0 ± 26.2b	125.4 ± 31.5b	103.7 ± 24.0b
*GNP1*–1/*NAL1*–1	49	158.4 ± 49.8	169.1 ± 52.3	130.7 ± 38.1
*GNP1*–6/*NAL1*–1	6	201.7 ± 40.2*	220.5 ± 27.3*	176.9 ± 40.4**

*N* the number of *xian* varieties, *GNP* grain number per panicle

All data are present as mean ± SD; Letters are ranked by Duncan test at *P* < 0.05; The *, ** denotes significance of Student’s *t* test at *P* < 0.05, *P* < 0.01, respectively

Based on estimates of variance components for four traits, TGW and GNP were controlled mainly by *V*_G_, whereas *V*_GEI_ was the main source for PN and GY in the introgression lines (ILs) with the Lemont background (LT-ILs) and the Teqing background (TQ-ILs) (Additional file 9: Table S3), indicating that PN and GY were highly environment-dependent, i.e., the two-gene combination had strong genotype by environment interaction on PN and GY (Wang et al. 2014). In LT-ILs and TQ-ILs, the *GNP1* and *NAL1* haplotypes were classified as strong or weak functional haplotypes based on their comparative performance in near-isogenic backgrounds (Xu et al. 2015; Wu et al. 2016). The Lemont carried the strong functional haplotype *NAL1*–4 (Xu et al. 2015), but a weak functional haplotype, *GNP1*–3, at *GNP1*. In contrast, the Teqing carried the weak functional haplotype *NAL1*–5, but a strong functional haplotype, *GNP1–*9, at *GNP1* (Wu et al. 2016). The 194 LT-ILs were classified into the following four types according to their genotypes in the *GNP1* (RM227–RM85) and *NAL1* (RM317–RM255) intervals: *GNP1*^W^*NAL1*^W^ (weak alleles at *GNP1* and *NAL1*), *GNP1*^S^*NAL1*^W^ (strong allele at *GNP1*, but a weak allele at *NAL1*), *GNP1*^W^*NAL1*^S^ (weak allele at *GNP1*, but a strong allele at *NAL1*), and *GNP1*^S^*NAL1*^S^ (strong alleles at *GNP1* and *NAL1*). We observed that the GNP of the germplasms with *GNP1*^S^*NAL1*^S^, *GNP1*^W^*NAL1*^S^, or *GNP1*^S^*NAL1*^W^ was higher than that of the germplasms with *GNP1*^W^*NAL1*^W^. Interestingly, the ILs carrying *GNP1*^S^*NAL1*^S^ had a higher GNP than the lines with *GNP1*^S^*NAL1*^W^ or *GNP1*^W^*NAL1*^S^ (15.7%–16.4% and 26.3%–38.9% higher, respectively) (Table 3). Moreover, the germplasms with *GNP1*^S^*NAL1*^S^ had a better average grain yield per plant (GYP) than the germplasms with *GNP1*^W^*NAL1*^S^ in Beijing and Sanya (24.2% and 18.7% higher, respectively) because of the substantially higher GNP associated with *GNP1*^S^*NAL1*^S^. However, the germplasms with *GNP1*^S^*NAL1*^S^ had a GYP that was similar to that of the germplasms with *GNP1*^S^*NAL1*^W^ because of a slightly lower grain weight and EPN (Table 3). In the TQ-ILs, the GNP was higher with *GNP1*^S^*NAL1*^S^ than with *GNP1*^S^*NAL1*^W^, which was similar to the results for the LT-ILs (Table 3). These findings suggested that in the *xian* and *geng* genetic backgrounds, the germplasms carrying both *GNP1* and *NAL1* had a considerably higher GNP than the germplasms carrying only *GNP1* or *NAL1*. This was inconsistent with the GYP data because of a decrease in the grain weight and EPN. Specifically, *GNP1*^S^*NAL1*^S^ and *GNP1*^S^*NAL1*^W^ resulted in a similar GYP that was significantly higher than that of *GNP1*^W^*NAL1*^S^.

**Table 3:** Grain yield and their related traits associated with the combination lines of *NAL1* and *GNP1*

GB	Regions	*GNP1*	*NAL1*	N	EPN	TGW	GNP	GYP
Lemont	Beijing	S	S	17	9.0 ± 1.8a	21.1 ± 2b	196.1 ± 42.3a	29.3 ± 9.4a
		S	W	14	9.6 ± 2.1a	22.3 ± 3.1ab	168.5 ± 37.9b	29.3 ± 10.5a
		W	S	151	8.8 ± 1.5a	22.2 ± 2ab	141.2 ± 21.2c	23.6 ± 5.9b
		W	W	12	9.9 ± 1.2a	24.2 ± 2.1a	114.6 ± 25.8d	18.5 ± 2.6c
	Sanya	S	S	17	11.9 ± 2b	22.1 ± 2.1b	220.7 ± 45.7a	47.6 ± 13a
		S	W	14	14.1 ± 2.7a	22.7 ± 2.8b	190.7 ± 38.4b	47.3 ± 12.4a
		W	S	151	12.6 ± 2.9ab	22.1 ± 1.6b	174.8 ± 28.4b	40.1 ± 12.5b
		W	W	12	14.2 ± 2.8a	24.0 ± 2.0a	143.5 ± 34c	41.1 ± 12.7b
Teqing	Beijing	S	S	16	8.7 ± 1.3a	22.4 ± 2.2a	219.3 ± 35.7a	28.8 ± 5.8ab
		S	W	217	9.4 ± 1.4a	22.0 ± 2.0a	195.8 ± 32.2b	30.2 ± 6.8a
		W	S	–	–	–	–	–
		W	W	19	8.9 ± 1.6a	22.8 ± 1.8a	164.6 ± 47.3c	25.6 ± 7.5b
	Sanya	S	S	16	11.6 ± 2.2a	23.8 ± 2.6a	241.8 ± 30.0a	47.0 ± 10.9a
		S	W	217	12.2 ± 2.7a	23.3 ± 2.0a	219.0 ± 33.4b	47.4 ± 14.0a
		W	S	–	–	–	–	–
		W	W	19	12.5 ± 2.7a	23.8 ± 2.2a	188.5 ± 33.2c	45.5 ± 11.5a

*GB* genetic background, *W* weak functional lines (Teqing allele of *NAL1*, Lemont allele of *GNP1*), *S* strong functional lines (Lemont allele of *NAL1*, Teqing allele of *GNP1*) (Xu et al. 2015; Wu et al. 2016), *N* the number of lines, *EPN* effective panicle number per plant, *TGW* thousand grain weight (g), *GNP* grain number per panicle, *GYP* grain yield per plant (g), theoretical yield in Sanya, actual yield in Beijing; All data are present as mean ± SD; Letters are ranked by Duncan’s test at *P* < 0.05

**Fig. 3:**
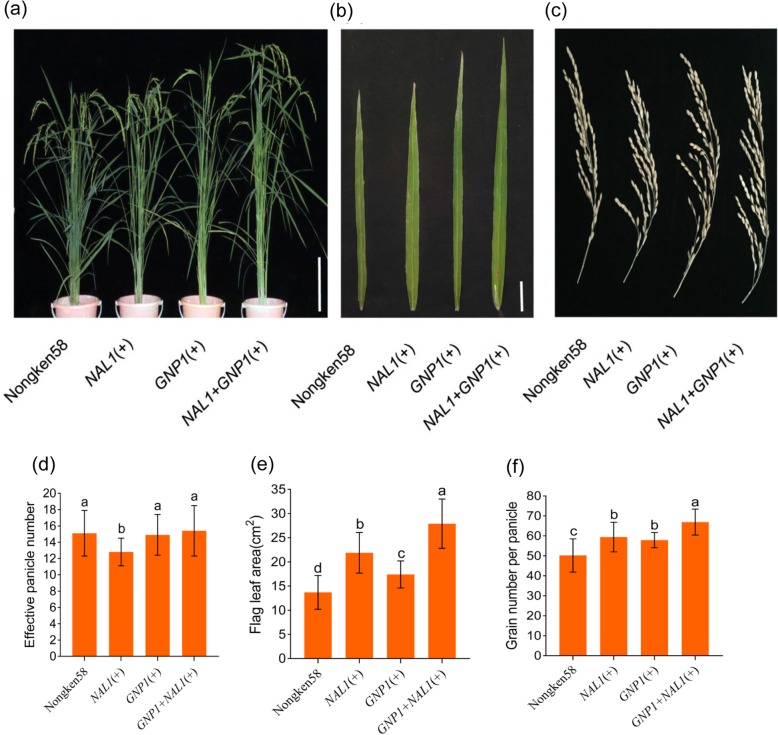
Performance of three homozygous transgenic lines in the T_3_ generation under nature conditions. **a**–**c** Cross morphologies (**a**) flag leaf morphologies (**b**) panicle morphologies (**c**) of the lines with *NAL1* or *GNP1*, the lines carrying both *NAL1* and *GNP1*, and Nongken58 (transgenic control). (+) indicate transgene-positive. **d**–**f** Effective panicle number (**d**) flag leaf area (**e**) grain number per panicle (**f**) are compared the lines with either *NAL1* or *GNP1* with the lines carrying both *NAL1*. Error bars indicate SD. Letters on histograms (a, b, and c) are ranked by Duncan’s test at *P* < 0.05. Scale bars represent 10 cm (a) or 2 cm (b)

To further clarify the combined effects of *GNP1* and *NAL1* on the GYP in the same background, we compared the grain yield-related traits of Nongken58 (transgenic control, carrying haplotype *NAL1*–5 of *NAL1* and *GNP1*–4 of *GNP1*) with those of homozygous transgenic lines with *GNP1* derived from the high-yielding *xian* cultivar Teqing (with a strong functional *GNP1* haplotype), *NAL1* derived from the *geng* cultivar Lemont (with a strong functional *NAL1* haplotype), or both *GNP1* and *NAL1*. Relative to the corresponding values for Nongken58, the *GNP1*-carrying transgenic plants had a significantly higher GNP as well as a substantially higher PH and flag leaf length (FLL) (Fig. 3, Table 4). Additionally, the *NAL1*-positive transgenic plants had a significantly higher GNP, FLW, FLL, and PH, with a significantly lower EPN (Fig. 3, Table 4). Interestingly, compared with the *GNP1-* and *NAL1-*carrying transgenic plants, the transgenic plants with both *GNP1* and *NAL1* had a significantly higher GNP (15.5%–25.4% and 11.6%–15.9% higher, respectively) and PH (6.5%–21.0% and 8.6%–17.8% higher, respectively), but a significantly lower thousand grain weight (TGW) (3.1%–3.9% and 7.5%–7.6% lower, respectively) across 3 years (Table 4). The GYP of the transgenic plants with both *GNP1* and *NAL1* was 5.7%–9.0% higher than that of the *GNP1*-carrying transgenic plants because of a large increase in the GNP. Moreover, the combination of *GNP1* and *NAL1* resulted in a GYP that was 8.3%–12.3% higher than that of *NAL1* alone because of a significant increase in the GNP and EPN (19.3%–21.7% higher) (Table 4).

**Table 4:** Performance of three homozygous transgenic lines under nature conditions

Generation	Genotype	FLL	FLW	PH	EPN	GNP	TGW	GYP	+% CK	+% *NAL1*	+% *GNP1*
T_2_	Nongken58	21.3b	1.0b	69.4c	19.0a	58.2c	–	–			
	*NAL1*(+)	24.5ab	1.3a	86.1b	15.7b	73.7b	–	–			
	*GNP1*(+)	27.1a	1.0b	83.2b	18.3a	68.1b	–	–			
	*GNP1* + *NAL1*(+)	28.6a	1.3a	93.5a	19.1a	85.4a	–	–			
T_3_	Nongken58	18.3b	1.0b	69.7c	15.1a	50.2c	27.1a	434.7			
	*NAL1*(+)	22.5a	1.3a	81.6b	12.8b	59.4b	27.5a	446.3	2.7		
	*GNP1*(+)	23.2a	1.0b	79.4b	14.9a	57.9b	26.2ab	460.1	5.8		
	*GNP1* + *NAL1*(+)	26.6a	1.4a	96.1a	15.4a	66.9a	25.4b	501.4	15.3	12.3	9.0
T_4_	Nongken58	23.3b	1.2b	96.0c	13.5a	70.7c	26.2a	530.3			
	*NAL1*(+)	30.6a	1.5a	107.6b	10.9b	90.6b	26.8a	553.9	4.5		
	*GNP1*(+)	32.4a	1.2b	110.6b	12.6ab	86.2b	25.8ab	567.2	7.0		
	*GNP1* + *NAL1*(+)	34.6a	1.5a	117.8a	13.0a	101.1a	24.8b	599.7	13.1	8.3	5.7

(+) indicate transgene-positive, *CK* Nongken58, *GNP* grain number per panicle, *EPN* effective panicle number per plant, *TGW* thousand grain weight (g), *FLL* flag leaf length (cm), *FLW* flag leaf width (cm), *PH* plant height (cm), *GYP* grain yield per plant (g/m^2^)

Letters are ranked by Duncan’s test at *P* < 0.05

## Discussion

### *GNP1* and *NAL1* are Important for the Natural Variation in Rice Grain Production

In the rice germplasms investigated in this study, *NAL1* was the most important locus for the GNP in both the *xian* and *geng* genetic backgrounds. These results were consistent with the findings of earlier studies involving near isogenic lines or transgenic rice lines carrying various alleles in diverse backgrounds (Fujita et al. 2013; Takai et al. 2013; Zhang et al. 2014; Xu et al. 2015; Yano et al. 2016). In previous investigations, sequence comparisons revealed that the *NAL1* allele from *xian* and *geng* rice cultivars had relatively conserved differences in the coding region (mainly one single-base substitution), which may differentially affect the development of multiple traits related to leaf type, plant type, and panicle type (Fujita et al. 2013; Takai et al. 2013; Zhang et al. 2014; Xu et al. 2015). The single-base substitution located at amino acid position 233 in the trypsin-like serine and cysteine protease domain lead to the amino acid variation (arginine and histidine). The *geng* cultivars Nipponbare, Koshihikari, and Lemont, which have identical superior *NAL1* alleles, carry the *NAL1*–4 haplotype. The introgression of the *NAL1*–4 alleles from *geng* rice germplasms into *xian* rice cultivars 9311, Takanari, and Teqing increased the GYP (Zhang et al. 2014), the leaf chlorophyll content (Zhang et al. 2014), the FLW (Xu et al. 2015), but decreased the photosynthetic rate per area (Takai et al. 2013). Taguchi-Shiobara et al. (2015) reported that the flag leaf width in transgenic plants of *NAL1* varied depending on the ratio of an A allele (histidine-type, H-type) to G allele (arginine-type, R-type). In 198 panels, accessions carrying *NAL1*-P3 protein haplotype had more GNP in 2014 and 2016, and larger FLW in 3 years compared with those carrying *NAL1*-P2(Additional file 8: Figure S6d–e). The difference in the one amino acid variation (histidine/ arginine) in the third exon was also observed between the two protein types (Additional file 8: Figure S6a) The H-type variation decreases the EPN, but increases the GNP and the FLW relative to the corresponding levels due to the R-type variation in the *geng* genetic background (Yano et al. 2016), which is highly consistent with our results obtained with the 3 K RGP *geng* subpopulation (Wang et al. 2018) in the Rice SNP-Seek Database (Additional file 10: Figure S7). In the present study, a SNP (S_2452) in the third exon was also significantly associated with the GNP and FLW (Table 1). Thus, we presumed that the nucleotide variation (A/G) in the third exon is critical for determining the GNP and FLW. Interestingly, the GNP and FLW differed between the germplasms with *NAL1*–2 or *NAL1*–6 in the *geng* subpopulation, despite the lack of an amino acid variation (R/H) and the insertion of a retrotransposon. A sequence comparison indicated that *NAL1*–2 and *NAL1*–6 differed because of a single-base InDel (S_1672) in the second intron and four nucleotide variations in the 3′ UTR (Fig. 2a). Additionally, the panicle *NAL1* expression levels significantly differed between the two haplotypes (Additional file 7: Figure S5b). Thus, we speculated the variability in the GNP between *NAL1*–2 and *NAL1*–6 might be due to the *NAL1* expression level and/or the single-base InDel (S_1672) significantly associated with the GNP in the 198 analyzed germplasms (Table 1). Interestingly, even with a lack of an R/H variation encoded in the third exon, a retrotransposon insertion, and expression level differences (Additional file 7: Figure S5c), differences in the GNP were still observed between *NAL1*–2 and the other two major *xian* haplotypes (*NAL1*–1 and *NAL1*–3) in the *xian* genetic background. A sequence comparison revealed that *NAL1*–2 and the other two haplotypes (*NAL1*–1 and *NAL1*–3) differed regarding the presence of previously reported nucleotide variations in the fifth exon resulting in two amino acid substitutions (Takai et al. 2013; Fujita et al. 2013; Zhang et al. 2014). In 198 accessions, accessions with *NAL1*-P1 exhibited significantly more GNP and larger FLW than those with *NAL1*-P2 in *xian* subpopulation (Additional file 8: Figure S6b–c). The difference in the two nucleotide variations in the fifth exon was also observed between the two protein types (Additional file 8: Figure S6a). Thus, we speculated these nucleotide differences in the fifth exon also influence the GNP. Additionally, grain number is also likely affected by other factors, including gene interactions or the genetic background effects, with consequences for the plant characteristics and yield.

A previous study indicated that *GNP1* increases the number of grains likely because of promoter sequence variations that alter *GNP1* expression (Wu et al. 2016). In the present study, a sequence comparison confirmed that the *GNP1* allele in the rare *GNP1*–9 haplotype, which is present in only four *xian* germplasms, is identical to that in Teqing with the superior *GNP1* haplotype described by Wu et al. (2016). Therefore, the Teqing allele is likely relatively uncommon. Additionally, two previously reported multiple-base deletions (I_1611 and I_1740) in the promoter region were observed in the major haplotype *GNP1*–5 and in three rare haplotypes (*GNP1*–9, *GNP1*–13, and *GNP1*–14) (Fig. 1a). In contrast, there was no significant difference in the GNP between *GNP1*–5 and the other three major haplotypes (*GNP1*–1, *GNP1*–2, and *GNP1*–6), even though these haplotypes include the two multiple-base sequence variations in the promoter. Interestingly, there were significant differences in the GNP between *GNP1*–6 and *GNP1*–1 within the *xian* subpopulation, despite the absence of the two multiple-base sequence differences in the promoter region (Fig. 1e). In addition, no significant difference in GNP was observed between two protein types in 3 years except in 2014 (Additional file 6: Figure S4b). To determine whether the sequence variability between the *GNP1*–6 and *GNP1*–1 promoter regions influences *GNP1* expression and contributes to the differences in the GNP, we analyzed the *GNP1* expression levels in the young panicles of the germplasms carrying *GNP1*–6 or *GNP1*–1. The *GNP1* transcript abundance in panicles was considerably greater in germplasms with *GNP1*–6 than in germplasms with *GNP1*–1 (Additional file 6: Figure S4a). Furthermore, we used PromPredict to predict the possible regulatory regions (Morey et al. 2011). Six potential promoter intervals, including one multiple-base deletion (I_1740) were identified in the promoter region (Fig. 1a). A single-base sequence variation (S_397) in the promoter contributed to the differences in the GNP and PH according to a gene-based association analysis (Table 1). However, the single-base alteration was not detected between Teqing and Lemont (Wu et al. 2016). These results implied that the nucleotide variation in the promoter region influences *GNP1* expression, thereby affecting the GNP and PH.

### Artificial Selection Patterns of *NAL1* and *GNP1*

A comparison of the *NAL1* sequences from the 198 *O. sativa* germplasms and the eight *O. rufipogon* germplasms revealed the *NAL1*–8 haplotype from the *Xian* haplogroup showed an allele that is identical to that of the wild variety WR4 (Fig. 2b), suggesting that it might be the original *xian* haplotype. Additionally, arginine was identified at amino acid position 233 in the *NAL1* of all eight germplasms of *O. rufipogon* (Fig. 2a), from which *O. sativa* originated. Accordingly, the R-type allele appears to be the ancestral form, from which the H-type allele was derived. Moreover, our data indicated that the R-type allele has been retained in all wild rice germplasms as well as in 98.6% of the *xian* and 58.1% of the *geng* germplasms among the 198 analyzed germplasms (Additional file 11: Table S4) during the evolution across large geographical distances (Additional file 12: Figure S8a). These observations imply the R-type alleles have a competitive advantage over the H-type alleles under natural conditions. Furthermore, we clarified the *NAL1* allele variation (R/H) based on the 3 K RGP database (Wang et al. 2018). We determined that 98.2% of the *xian* and 25.1% of the *geng* germplasms carry the R-type allele, whereas only 1% of the *xian* and 73.4% of the *geng* germplasms carry the H-type allele from 3 K RGP (Additional file 11: Table S4). These results suggest that the differences in the *NAL1* allelic frequency might be related to the *xian*–*geng* differentiation. In the present study, the H-type allele appeared to be introgressed into a few *xian* landraces (2 of 146 among the 198 analyzed germplasms; 18 of 1785 in 3 K RGP). These landraces include a defined allele comprising the *geng*-like DNA flanking *NAL1* in the *xian* background, which was likely the result of naturally occurring crosses and the subsequent introgression during domestication after the emergence of the H-type allele in the *geng* subpopulation. A 5895-bp retrotransposon insertion in the second exon of *NAL1* was detected only in haplotype *NAL1*–4 of *geng*, suggesting that germplasms with haplotype *NAL1*–4 might be from the same ancestor. The *NAL1*–4 haplotype was present in 18 germplasms widely distributed in nine countries on five continents (Asia, Australia, Europe, Africa, and South America) (Additional file 12: Figure S8a). A phylogenetic network revealed that a retrotransposon insertion converted *NAL1*–10 to *NAL1*–4 (Fig. 2b).

The *GNP1* haplotype of *O. sativa* ssp. *geng*, which is highly conserved (i.e., only one single-base variation), may have evolved from a unique ancient *O. rufipogon* germplasm. Additionally, the *GNP1* nucleotide diversity in the *geng* landraces (π = 0.00085) was 68% lower than that of *O. rufipogon* (π = 0.00264). In contrast, the *GNP1* nucleotide diversity in the *xian* landraces (π = 0.00255) decreased by only 3% (Additional file 4: Table S2). The nucleotide diversity of the *geng GNP1* was similar to that of 111 randomly chosen gene fragments (π = 0.00111) (Caicedo et al. 2007), suggesting that the lower nucleotide diversity in *geng* than in *xian* or the wild progenitor was due to a genetic bottleneck effect rather than a selective sweep (Nagano et al. 2005).

### Favorable Haplotypes/Alleles for Increasing the GNP in Rice

At the single-gene level, we identified favorable haplotypes for the two genes influencing the GNP. The *NAL1*–3 and *NAL1*–1 haplotypes as well as the *GNP1*–6 haplotype increased the GNP in the *xian* subpopulation. Therefore, these superior haplotypes may be ideal for increasing the GNP in *xian* rice cultivars. In the *geng* subpopulation, some cultivars with the superior haplotypes *NAL1*–2 and *NAL1*–4 (e.g., Lemont and Nipponbare) that resulted in increased grain number (sink-related trait) and wider flag leaves (source-related trait) may be the most appropriate germplasms for the molecular breeding of super high-yielding rice varieties with ideal plant characteristics and a balanced sink and source relationship. The findings described herein may be relevant for introducing superior haplotypes of a single gene into a single variety with the ideal genotype.

In a previous study, we determined that the rice grain yield of a near-isogenic line with the superior *NAL1* allele from Lemont in a Teqing genetic background was 3.2%–3.8% higher than that of Teqing in three environments (Xu et al. 2015). Teqing is a well-known super-high-yielding and widely adaptable *xian* variety developed in China in 1984, with a grain yield that is 30% higher than that of Lemont under the growth conditions in the southern USA (Li et al. 1998). In other words, the superior *NAL1* allele from the low-yielding Lemont germplasm can further increase the grain yield of high-yielding varieties such as Teqing. Previous studies on the molecular basis of complex traits proved that even though the germplasms themselves do not exhibit the target trait characteristics, they may still harbor favorable genes for increasing yield (He et al. 2006), salt tolerance (Zang et al. 2008), and drought tolerance (Wang et al. 2012). Therefore, to enhance quantitative traits, such as grain yield, the favorable alleles in some low-yielding varieties may be relevant for rice breeding programs.

Regarding *GNP1*, there were no significant differences in the GNP resulting from the *GNP1*–3 and *GNP1*–4 *geng* haplotypes. The superior *GNP1*–9 (Teqing) and *GNP1*–6 haplotypes were respectively detected in only 2% and 4% of the 198 germplasms (Fig. 1a). Therefore, we propose the superior *GNP1* haplotype has no significant effects in the *geng* subpopulation and is rare in the *xian* background of the 198 analyzed cultivated rice germplasms. In a genome-wide association study of 3000 rice accessions, no significant SNP was identified in the *GNP1* chromosomal regions affecting the GNP, implying the favorable *GNP1* allele is relatively uncommon. The near-isogenic line NIL-*GNP1*^TQ^ in the Lemont genetic background, which differs from Lemont only in an approximately 66.1-kb region containing *GNP1* derived from Teqing, reportedly produces 5.7%–9.6% more grain yield than Lemont (Wu et al. 2016). These results suggest that the rare Teqing superior *GNP1* allele may be useful for breeding high-yielding rice varieties. Many superior alleles mediating grain number, grain size, and grain weight, including *OsSPL14* (Jiao et al. 2010), *GS2* (Hu et al. 2015), *OsglHAT1* (Song et al. 2015), are relatively rare at the natural population scale, but may be applicable for increasing rice yield in breeding programs (Jiao et al. 2010; Hu et al. 2015; Kim et al. 2018). Most parental inbred lines have only a few rare superior alleles, whereas high-yielding hybrid varieties possess several of these alleles (Huang et al. 2015). Increasing diversity via *xian* × *geng* crosses, which may enable the relatively rapid introduction of low-frequency superior alleles in specific lines, should be pursued in future studies involving hybrid rice breeding (Huang et al. 2015).

### Two-Gene Combinations for Increasing the GNP and GYP in Rice

In this study, we proved that combining *NAL1* and *GNP1* increases the GNP in different genetic backgrounds. Specifically, the ILs carrying the strong *NAL1* and *GNP1* alleles (*GNP1*^S^*NAL1*^S^) in the Lemont genetic background had a higher GNP than the lines with only one strong allele (*GNP1*^W^*NAL1*^S^ or *GNP1*^S^*NAL1*^W^) (Table 3). In the Teqing background, the GNP resulting from *GNP1*^S^*NAL1*^S^ was significantly higher than that due to *GNP1*^S^*NAL1*^W^ (Table 3). Moreover, the GNP of the transgenic lines carrying the favorable Teqing allele of *GNP1* and Lemont allele of *NAL1* was significantly higher than the GNP of transgenic lines carrying only *GNP1* or *NAL1* (Table 4). These results suggest these two genes may have a synergistic interactive effect on the regulation of grain number.

Compared with the *NAL1* transgenic plants, the lines carrying *NAL1* and *GNP1* had a significantly higher flag leaf area (source-related trait), GNP (sink-related trait), and EPN, which increased the GYP by an average of 8.3%–12.3% (Fig. 3, Table 4). Similarly, the transgenic lines with both *NAL1* and *GNP1* had a higher flag leaf area and GNP than the transgenic plants with *GNP1* alone, which resulted in an average increase in the GYP of 5.7%–9.0% (Fig. 3, Table 4). Our results imply that the combined effects of *NAL1* and *GNP1* may enhance the source–sink relationship, thus resulting in increased the yield in *geng* rice cultivar although they were also slight taller than single transgenic lines. Therefore, exploiting and pyramiding superior haplotypes (e.g., *NAL1* from Lemont and *GNP1* from Teqing) may enable the breeding of super-high-yielding rice varieties with optimized plant characteristics and source–sink relationships.

## Conclusions

We identified 16 and 14 haplotypes for *NAL1* and *GNP1*, respectively. The *NAL1* gene had the strongest effects on GNP in *xian* and *geng* subpopulations while *GNP1* had no significant effects in the *geng* subpopulation and was rare in the *xian* background. The transgenic lines with both genes exhibited higher GNP and grain yield than the transgenic lines with *GNP1* or *NAL1*. The two genes combined in the introgression lines in Lemont background had favorable effects on the GNP. These results should contribute to rice breeding for high yield through pyramiding *GNP1* and *NAL1*.

## Methods

### Plant Materials and Phenotyping

A total of 198 *O. sativa* germplasms varying regarding the GNP, FLW, and PH from 28 countries were obtained from the China National Crop Genebank of the Chinese Academy of Agricultural Science and the International Rice Research Institute. An additional 8–10 common wild rice (*O. rufipogon*) germplasms were obtained from the Guangxi Academy of Agricultural Sciences, Guangxi, China. Basic information for each germplasm is provided in Additional file 3: Table S1 and Additional file 13: Table S5. All germplasms were grown under normal field conditions at the following two locations over 3 years: Nanning (22.1°N, 107.5°E) in Guangxi province in 2014 and 2015 and Shenzhen (22.6°N, 114.1°E) in 2016. The field trial involved two rows of 12 plants each, with the rows separated by 25 cm and the plants in each row separated by 17 cm. The analysis was completed with three replicates. The fields were managed according to the local standard practices. At the heading stage, the FLW was measured for the main stem of each plant. At maturity, the GNP and PH of eight plants were measured.

Two sets of reciprocal ILs comprising 201 lines (32 BC_2_F_8_, 123 BC_3_F_7_, and 46 BC_4_F_6_) in the Lemont background (LT-ILs) and 252 lines (133 BC_2_F_8_, 96 BC_3_F_7_, and 23 BC_4_F_6_) in the Teqing background (TQ-ILs) were generated from the consecutive backcrossing and several generations of selfing between the *xian* cultivar Teqing and the *geng* cultivar Lemont (Mei et al. 2006). These lines were grown in Sanya (18.3°N, 109.3°E) in the winter of 2014 and in Beijing (40.2°N, 116.2°E) in the summer of 2015. At maturity, the EPN, TGW, GYP, and GNP were determined. After eliminating the lines with missing phenotypic data, the remaining lines were used for analyzing the effects of *NAL1* and *GNP1* on the EPN, GNP, TGW, and GYP in the two (LT and TQ) backgrounds.

### DNA Extraction, PCR Amplification, Gene Cloning, and Sequencing

Fresh leaves were collected from the field-grown plants for a genomic DNA extraction with the cetyltrimethylammonium bromide method (Murray and Thompson 1980). The primers used to amplify the promoter, 5′ UTR, exon, intron, and 3′ UTR were designed based on the Nipponbare *GNP1* and *NAL1* alleles. The PCR was completed with KOD NEO DNA polymerase (Toyobo, Shiga, Japan) and a standard PCR protocol.

Because of the existence of heterozygous genotypes in *O. rufipogon*, the PCR product was ligated into the pGEM-T Easy Vector (Promega, USA) and then sequenced, after which one allele sequence was randomly selected. To ensure accuracy, the sequencing was performed twice with the ABI 3730 system at the Beijing Genomics Institute, China. Sequence contigs were assembled with the SEQUENCHER 4.1.2 program (Gene Codes Corporation, Ann Arbor, MI, USA). Details regarding the PCR primers and sequencing are listed in Additional file 14: Table S6.

### Analyses of DNA Sequences and Population Structures

The DNA sequences were aligned with the MUSCLE program (Edgar 2004) and manually adjusted with BIOEDIT (Hall 1999). The number of segregating sites, nucleotide diversity (π), Watterson’s estimator (θ), and Tajima’s D and Fu and Li’s D of the neutrality test were calculated with the DNASP program (version 5.0) (Librado and Rozas 2009). The sliding-window method was employed to analyze the polymorphisms in the *GNP1* and *NAL1* genome sequences, with a window size of 100 bp and a step size of 20 bp, in which pairwise insertions and deletions were removed with DNASP (version 5.0). The evolutionary history was inferred based on the neighbor-joining method of the MEGA5 program (Tamura et al. 2011). The phylogenetic network was constructed with Network 4.611 (Hans-Jurgen et al. 1999) according to the corresponding user guide.

For population structure analyses, 24,460 evenly distributed SNPs were sampled to calculate the population structure (Q). We used a model-based Bayesian clustering analysis method implemented with the STRUCTURE program (version 2.3.4) (Pritchard et al. 2000). The Q matrix derived from the principal components was calculated with a Perl script and SNPs filtered with Plink (Purcell et al. 2007). The K matrix (kinship matrix) was obtained from the results of the relatedness analysis according to the EMMA method in GAPIT, which is an R software package (Lipka et al. 2012).

To minimize the effects of environmental variations, the best linear unbiased predictors (BLUPs) for each germplasm phenotype over 3 years were calculated with the R package rrBLUP (version 4.3) (Endelman 2011). The SNPs with a minor allele frequency less than 0.05 and/or a missing data rate exceeding 20% were removed and the remaining high-quality SNPs within *NAL1* and *GNP1* were used to perform gene-based association analyses via the mixed linear model with Q and K. The analyses were performed with the TASSEL program (Bradbury et al. 2007).

### Gene Expression Analysis

Yong panicles were collected from five plants per germplasm at the panicle initiation stage for a subsequent gene expression analysis. Total RNA was extracted from the young panicles with the TRIzol reagent (Invitrogen), after which 2 μg total RNA was used as the template for a cDNA synthesis with SuperScript III reverse transcriptase (Invitrogen) in a final volume of 20 μl. A real-time PCR assay was performed with gene-specific primers (Additional file 14: Table S6) and a 7500 Real-Time PCR system (Applied Biosystems, Carlsbad, CA, USA) according to the manufacturer’s instructions. The rice *Actin* gene was used as the internal control. Each sample was analyzed with three technical replicates.

### Complementation Test

For the *NAL1* complementary test, a 13.8-kb DNA fragment from 3424 bp upstream of the transcription start site to 1450 bp downstream of the termination site was amplified from Lemont. The sequence was then cloned into pCAMBIA1300 to produce the pCAMBIA1300-*NAL1* recombinant plasmid. For *GNP1*, the 5.5-kb full-length genomic DNA sequence from 3498 bp upstream of the transcription start site to 878 bp downstream of the termination site was amplified from Teqing. The PCR product was cloned into pCAMBIA1300 to generate the pCAMBIA1300-*GNP1* recombinant plasmid. Additionally, a 19.3-kb fragment comprising the 13.8-kb *NAL1* DNA fragment from Lemont and the 5.5-kb *GNP1* genomic DNA from Teqing was inserted into the same pCAMBIA1300 vector. Specifically, the 5.5-kb *GNP1* genomic sequence was amplified from Teqing with primers containing the *Sbf*I and *Pst*I sequences. The amplified fragment (5.5-kb) was digested with restriction enzymes and then inserted into pCAMBIA1300 at the *Sbf*I and *Pst*I sites to produce the pCAMBIA1300-*GNP1*–2 recombinant plasmid. The 13.8-kb *NAL1* fragment was removed from pCAMBIA1300-*NAL1* by a digestion with *BamH*I and *Sbf*I. The resulting *NAL1* fragment (13.8-kb) was inserted into the *BamH*I and *Sbf*I sites of pCAMBIA1300-*GNP1–*2 to generate the pCAMBIA1300-*GNP1* + *NAL1* recombinant plasmid. The three recombinant plasmids were separately introduced into *Agrobacterium tumefaciens* strain EHA105 cells for the subsequent transformation of a *geng* variety, Nongken58. The primer sets used for the PCR amplifications are listed in Additional file 14: Table S6.

The three transgenic lines and Nongken58 were planted with a randomized block design (with three replicates) at a spacing of 25 × 17 cm in Sanya (18.3°N, 109.3°E), Hainan province, China during December 2015–April 2016, February–June, 2017, and December 2017–April 2018, respectively. At the heading stage, the FLL, FLW, and PH were measured for the main stem of each plant. At maturity, eight plants were sampled and dried in an oven at 70 °C for 5 days, after which the EPN, GNP, and TGW were calculated. Additionally, plants were harvested from a 2 m^2^ area in each plot and then air dried before determining the GYP.

### Statistical Analysis

Differences in the phenotypic values and in the gene expression levels between the haplotypes (containing more than seven accessions) were examined by a one-way ANOVA or Student’s *t*-test. Duncan’s multiple range test was conducted to determine the significance of any differences (*P* < 0.05). The data analyses were performed with the SAS software. To dissect effect of genotype-by-environment interaction (GEI) on the tested traits, variance components were estimated using multiple-site analysis with all effects treated as random. Heritability across environments was computed using the estimated variance components as *V*_G_/(*V*_G_ + *V*_GEI_/*s* + *V*_e_/*sr*), where *V*_G_, *V*_GEI_, and *V*_e_ are the variances of genotype, GEI, and residual error, respectively, *s* is the number of environments, and *r* is the number of replicates. All analyses were conducted with the PBTools package (http://bbi.irri.org/products) developed by IRRI.

## Supplementary information

**Additional file 1: Figure S1.** Comparison of grain number per panicle, flag leaf width and plant height between *xian* and *geng* rice (*Oryza sativa* L.) subpopulations in 198 accessions. (a–c) Cyan and orange colors indicate *xian* and *geng*, respectively. The *, **, *** denotes significance of Student’ s *t* test at *P* < 0.05, *P* < 0.01, and *P* < 0.001, respectively.

**Additional file 2: Figure S2.** Population structure analyses of 198 accessions. (a) Scree plot from GAPIT showing the selection of PCs for association study. (b) PCA plot based on the screen plot in the rice diversity panel. (c) Scree plot from STRUCTURE showing the selection of Q. (d) Bayesian clustering of 198 accessions using STRUCTURE program.

**Additional file 3 : Table S1.** Details of the accessions investigated and two grain number gene haplotypes.

**Additional file 4 : Table S2.** Nucleotide diversity of *GNP1* and *NAL1* genes. S, segregation sites; π, average number of nucleotide differences per site between random two sequences; θ, Watterson estimator; D1, Tajima’s D; D2, Fu and Li’D; D3, Fu and Li’F.

**Additional file 5 : Figure S3.** Sliding-windows analysis of *GNP1* (a) and *NAL1* (b) nucleotide diversity in *O. sativa* ssp. *xian*, *O. sativa* ssp. *geng* and *O. rufipogon.* The *y*-axis represents nucleotide diversity (*π*), and the genomic structure is shown at the bottom, where the black boxes indicate exons and the white boxes indicate introns and other noncoding regions.

**Additional file 6 : Figure S4.** Protein diversity of *GNP1* (a). Comparison of grain number per panicle, and plant height between two protein types in *xian* subpopulations in 198 panel (b–c). Cyan and orange colors indicate *GNP1*-P1 and *GNP1*-P2, respectively. The *, **, *** denotes significance of Student’ s *t* test at *P* < 0.05, *P* < 0.01, and *P* < 0.001, respectively.

**Additional file 7 : Figure S5.** Expression level of *NAL1* and *GNP1* haplotypes in young panicle at the panicle initiation stage. (a) Expression level of *GNP1*–1 and *GNP1–6* in *xian* subpopulation. (b) Expression level of *NAL1*–2, *NAL1*–4 and *NAL1*–6 in *geng* subpopulation. (c) Expression level of *NAL1*–1, *NAL1*–2 and *NAL1*–3 in *xian* subpopulation. Error bars indicate SD; Letters are ranked by Duncan’s test at *P* < 0.05. The * denotes significance of Student’ s *t* test at *P* < 0.05.

**Additional file 8 : Figure S6.** Protein diversity of *NAL1* (a). Comparison of grain number per panicle, and flag leaf width among three protein types in *xian* and *geng* subpopulations, respectively (b–e). Cyan, orange, and lightpink colors indicate *NAL1*-P1, *NAL1*-P2 and *NAL1*-P3, respectively. The *, **, *** denotes significance of Student’ s *t* test at *P* < 0.05, *P* < 0.01, and *P* < 0.001, respectively.

**Additional file 9 : Table S3.** Variance components and heritabilty estimated by multiple-site analysis.

**Additional file 10 : Figure S7.** Comparison of grain number per panicle, flag leaf width and effective panicle number between R-type and H-type alleles located in the third exon of *NAL1* gene in 3 K panel in *xian* and *geng* subpopulation, respectively. ***, *P* < 0.001 (Student’ s *t* test).

**Additional file 11 : Table S4.** Distribution of protein mutation sites diversity at amino acid position 233 of *NAL1* gene. R, arginine; H, histidine.

**Additional file 12 : Figure S8.** Geographic distributions of different haplotypes of *NAL1* and *GNP1* among the 28 areas sampled.

**Additional file 13 : Table S5.** Details of the wild rice (*Oryza rufipogon*).

**Additional file 14 : Table S6.** Sequencing primers used in this study.

## Data Availability

All data generated or analyzed during this study are included in this published article and its supplementary information files.

## Abbreviations

EPN: Effective panicle number per plant
FLL: Flag leaf length
FLW: Flag leaf width
GNP: Grain number per panicle
H-type: Histidine-type
ILs: Introgression lines
LT-ILs: Introgression lines with the Lemont background
PH: Plant height
QTL: Quantitative trait loci
R-type: Arginine-type
TQ-ILs: Introgression lines with the Teqing background
