# Supplementary material for: Natural Sequence Variations and Combinations of GNP1 and NAL1 Determine the Grain Number per Panicle in Rice
Source: Rice (N Y). 2020 Feb 28;13:14. doi: 10.1186/s12284-020-00374-8 (PMC7048901; doi:10.1186/s12284-020-00374-8)
Supplement: Supplementary file 1 — Additional file 1: Figure S1. Comparison of grain number per panicle, flag leaf width and plant height between xian and geng rice (Oryza sativa L.) subpopulations in 198 accessions. (a–c) Cyan and orange colors indicate xian and geng, respectively. The *, **, *** denotes significance of Student’ s t test at P < 0.05, P < 0.01, and P < 0.001, respectively. [file 12284_2020_374_MOESM1_ESM.ppt]

## Slide 1
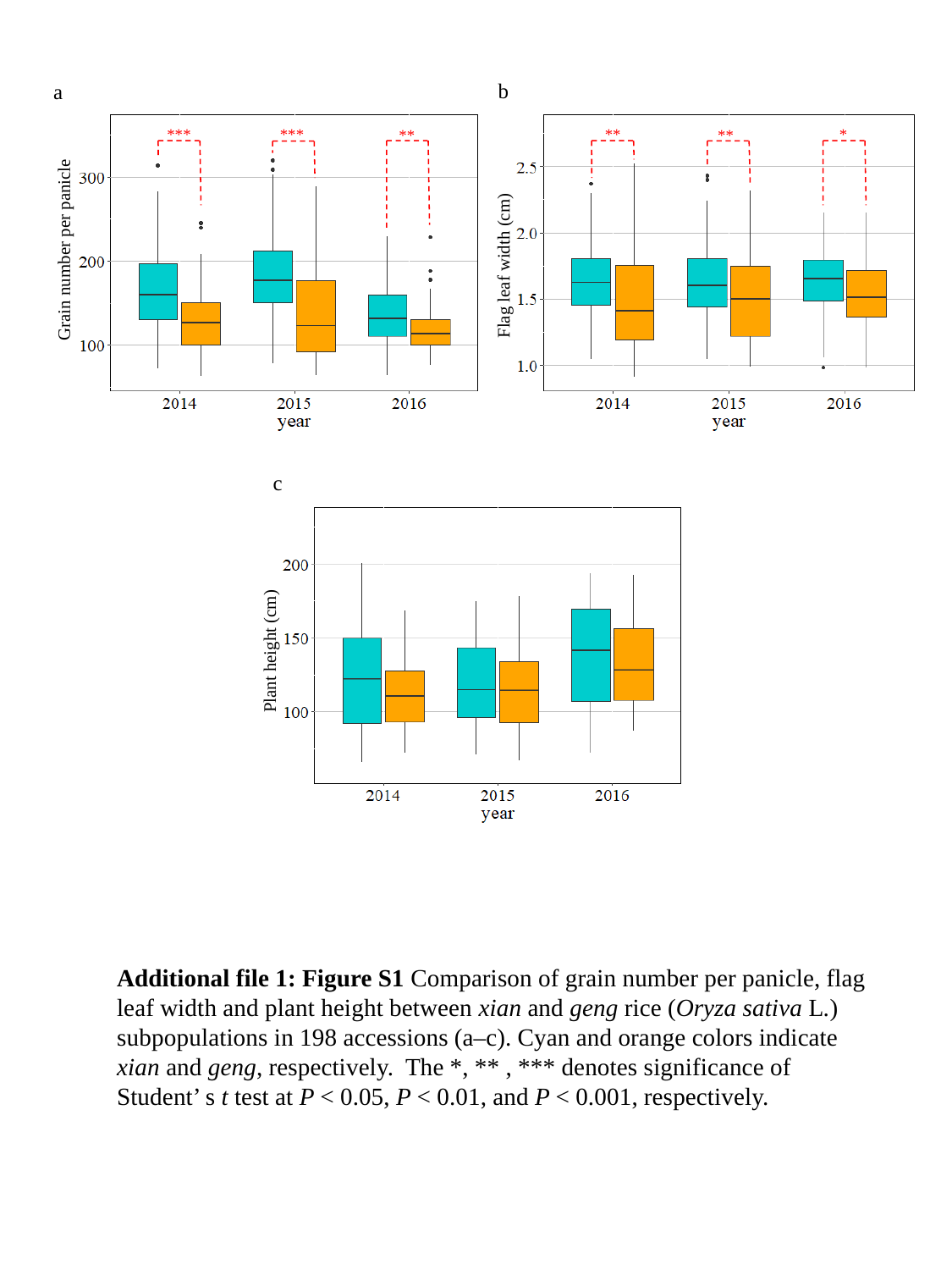

b
a
***
***
**
*
**
**
Grain number per panicle
Flag leaf width (cm)
c
Plant height (cm)
Additional file 1: Figure S1 Comparison of grain number per panicle, flag leaf width and plant height between xian and geng rice (Oryza sativa L.) subpopulations in 198 accessions (a–c). Cyan and orange colors indicate xian and geng, respectively. The *, ** , *** denotes significance of Student’ s t test at P < 0.05, P < 0.01, and P < 0.001, respectively.
