# Supplementary material for: Natural Sequence Variations and Combinations of GNP1 and NAL1 Determine the Grain Number per Panicle in Rice
Source: Rice (N Y). 2020 Feb 28;13:14. doi: 10.1186/s12284-020-00374-8 (PMC7048901; doi:10.1186/s12284-020-00374-8)
Supplement: Supplementary file 2 — Additional file 2: Figure S2. Population structure analyses of 198 accessions. (a) Scree plot from GAPIT showing the selection of PCs for association study. (b) PCA plot based on the screen plot in the rice diversity panel. (c) Scree plot from STRUCTURE showing the selection of Q. (d) Bayesian clustering of 198 accessions using STRUCTURE program. [file 12284_2020_374_MOESM2_ESM.pdf]

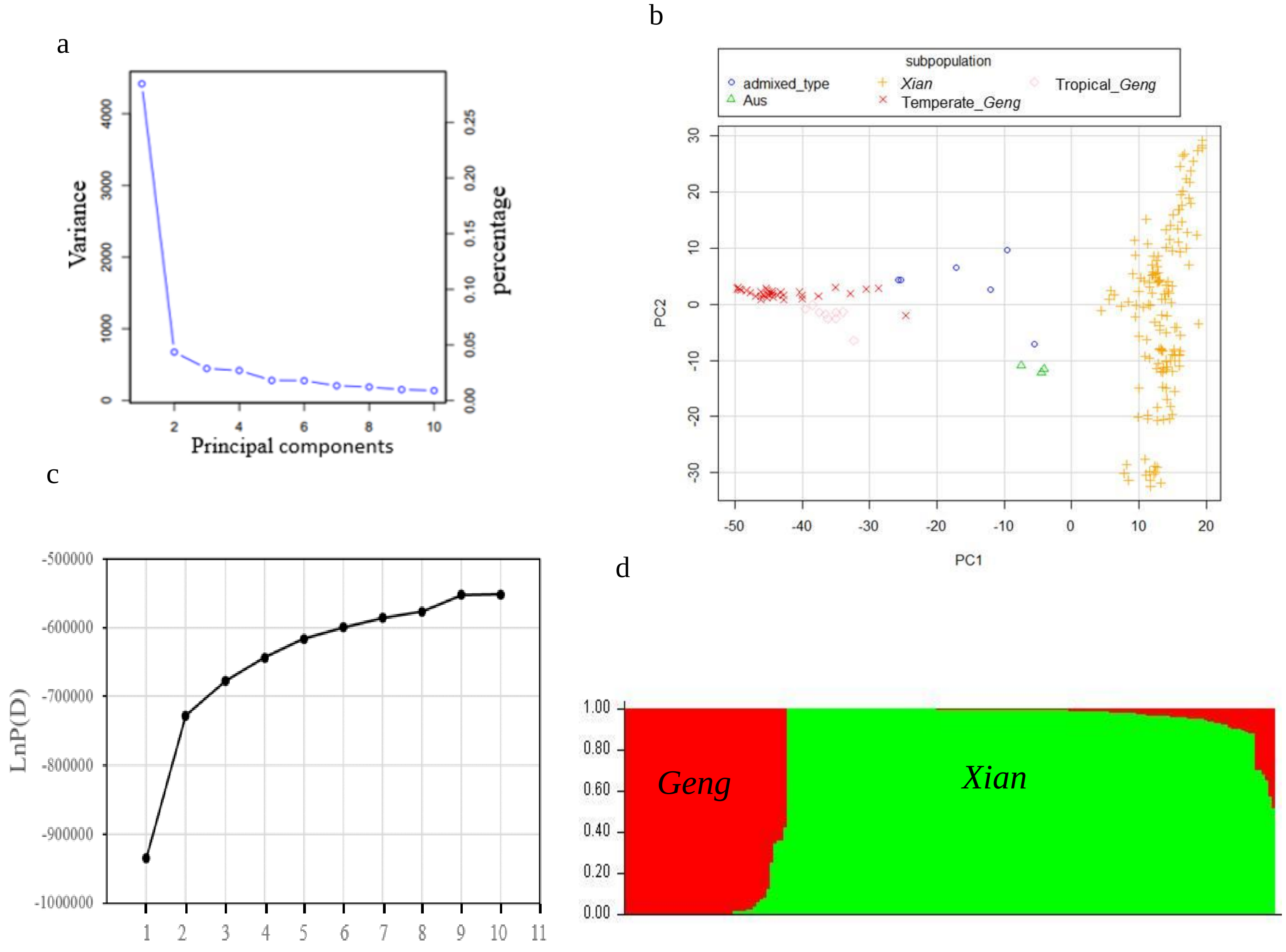

Additional file 2: Figure S2 Population structure analyses of 198 accessions. (a) Scree plot from GAPIT showing the selection of PCs for association study. (b) PCA plot based on the scree plot in the rice diversity panel. (c) Screen plot from STRUCTURE showing the selection of Q. (d) Bayesian clustering of 198 accessions using STRUCTURE program.
