# Supplementary material for: Natural Sequence Variations and Combinations of GNP1 and NAL1 Determine the Grain Number per Panicle in Rice
Source: Rice (N Y). 2020 Feb 28;13:14. doi: 10.1186/s12284-020-00374-8 (PMC7048901; doi:10.1186/s12284-020-00374-8)
Supplement: Supplementary file 4 — Additional file 4 : Table S2. Nucleotide diversity of GNP1 and NAL1 genes. S, segregation sites; π, average number of nucleotide differences per site between random two sequences; θ, Watterson estimator; D1, Tajima’s D; D2, Fu and Li’D; D3, Fu and Li’F. [file 12284_2020_374_MOESM4_ESM.docx]

**Additional file 4: Table S2** Nucleotide diversity of *GNP1* and *NAL1* genes

| Genes | Species(N) | S | π (10^-3^) | θ (10^-3^) | D1 | D2 | D3 |
| --- | --- | --- | --- | --- | --- | --- | --- |
| *GNP1*(3675bp) | All (208) | 57 | 2.42 | 2.70 | -0.31 | -1.33 | -1.05 |
|  | *O. sativa* (198) | 48 | 2.48 | 2.26 | 0.30 | -0.50 | -0.18 |
|  | *O. sativa* ssp. *xian* (146) | 46 | 2.55 | 2.27 | 0.37 | -0.20 | 0.05 |
|  | *O. sativa* ssp. *geng* (43) | 24 | 0.85 | 1.48 | -1.39 | -1.75 | -1.93 |
|  | *O. rufipogon* (10) | 38 | 2.64 | 3.68 | -1.32 | -1.21 | -1.41 |
| *NAL1* (10287bp) | All (206) | 47 | 1.66 | 1.81 | -0.25 | 0.28 | 0.06 |
|  | *O. sativa* (198) | 42 | 1.65 | 1.63 | 0.03 | 0.37 | 0.27 |
|  | *O. sativa* ssp. *xian* (146) | 41 | 1.14 | 1.67 | -0.95 | 0.41 | -0.20 |
|  | *O. sativa* ssp. *geng* (43) | 24 | 0.88 | 1.23 | -0.93 | -0.80 | -1.00 |
|  | *O. rufipogon* (8) | 12 | 1 | 1.05 | -0.26 | -0.21 | -0.24 |

S, segregation sites; π, average number of nucleotide differences per site between random two sequences; θ, Watterson estimator; D1, Tajima’s D; D2, Fu and Li’D; D3, Fu and Li’F.
