# Supplementary material for: Natural Sequence Variations and Combinations of GNP1 and NAL1 Determine the Grain Number per Panicle in Rice
Source: Rice (N Y). 2020 Feb 28;13:14. doi: 10.1186/s12284-020-00374-8 (PMC7048901; doi:10.1186/s12284-020-00374-8)
Supplement: Supplementary file 5 — Additional file 5 : Figure S3. Sliding-windows analysis of GNP1 (a) and NAL1 (b) nucleotide diversity in O. sativa ssp. xian, O. sativa ssp. geng and O. rufipogon. The y-axis represents nucleotide diversity (π), and the genomic structure is shown at the bottom, where the black boxes indicate exons and the white boxes indicate introns and other noncoding regions. [file 12284_2020_374_MOESM5_ESM.ppt]

## Slide 1
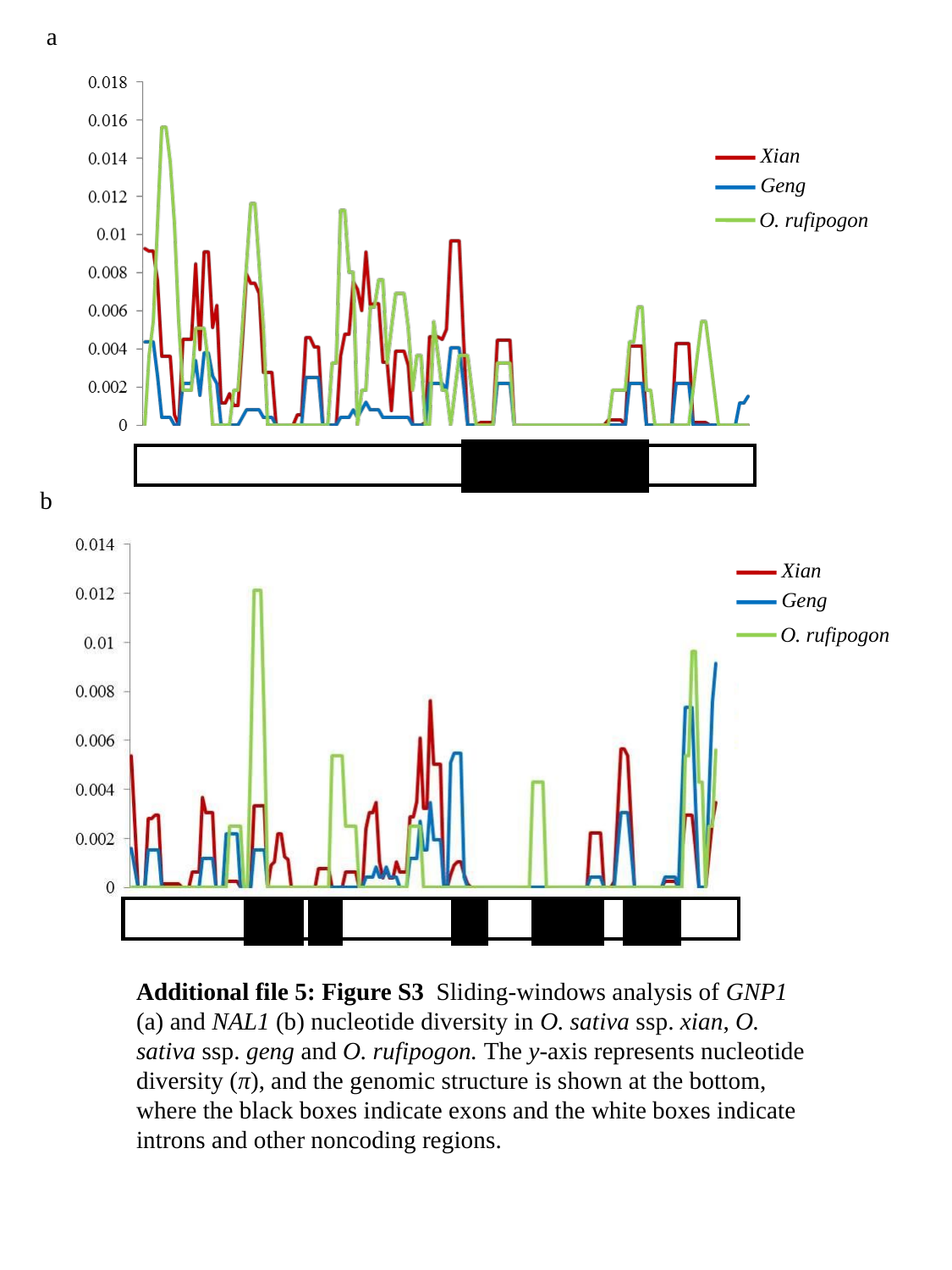

a
Xian
Geng
O. rufipogon
b
Xian
Geng
O. rufipogon
Additional file 5: Figure S3 Sliding-windows analysis of GNP1 (a) and NAL1 (b) nucleotide diversity in O. sativa ssp. xian, O. sativa ssp. geng and O. rufipogon. The y-axis represents nucleotide diversity (π), and the genomic structure is shown at the bottom, where the black boxes indicate exons and the white boxes indicate introns and other noncoding regions.
