# Supplementary material for: Natural Sequence Variations and Combinations of GNP1 and NAL1 Determine the Grain Number per Panicle in Rice
Source: Rice (N Y). 2020 Feb 28;13:14. doi: 10.1186/s12284-020-00374-8 (PMC7048901; doi:10.1186/s12284-020-00374-8)
Supplement: Supplementary file 6 — Additional file 6 : Figure S4. Protein diversity of GNP1 (a). Comparison of grain number per panicle, and plant height between two protein types in xian subpopulations in 198 panel (b–c). Cyan and orange colors indicate GNP1-P1 and GNP1-P2, respectively. The *, **, *** denotes significance of Student’ s t test at P < 0.05, P < 0.01, and P < 0.001, respectively. [file 12284_2020_374_MOESM6_ESM.pptx]

## Slide 1
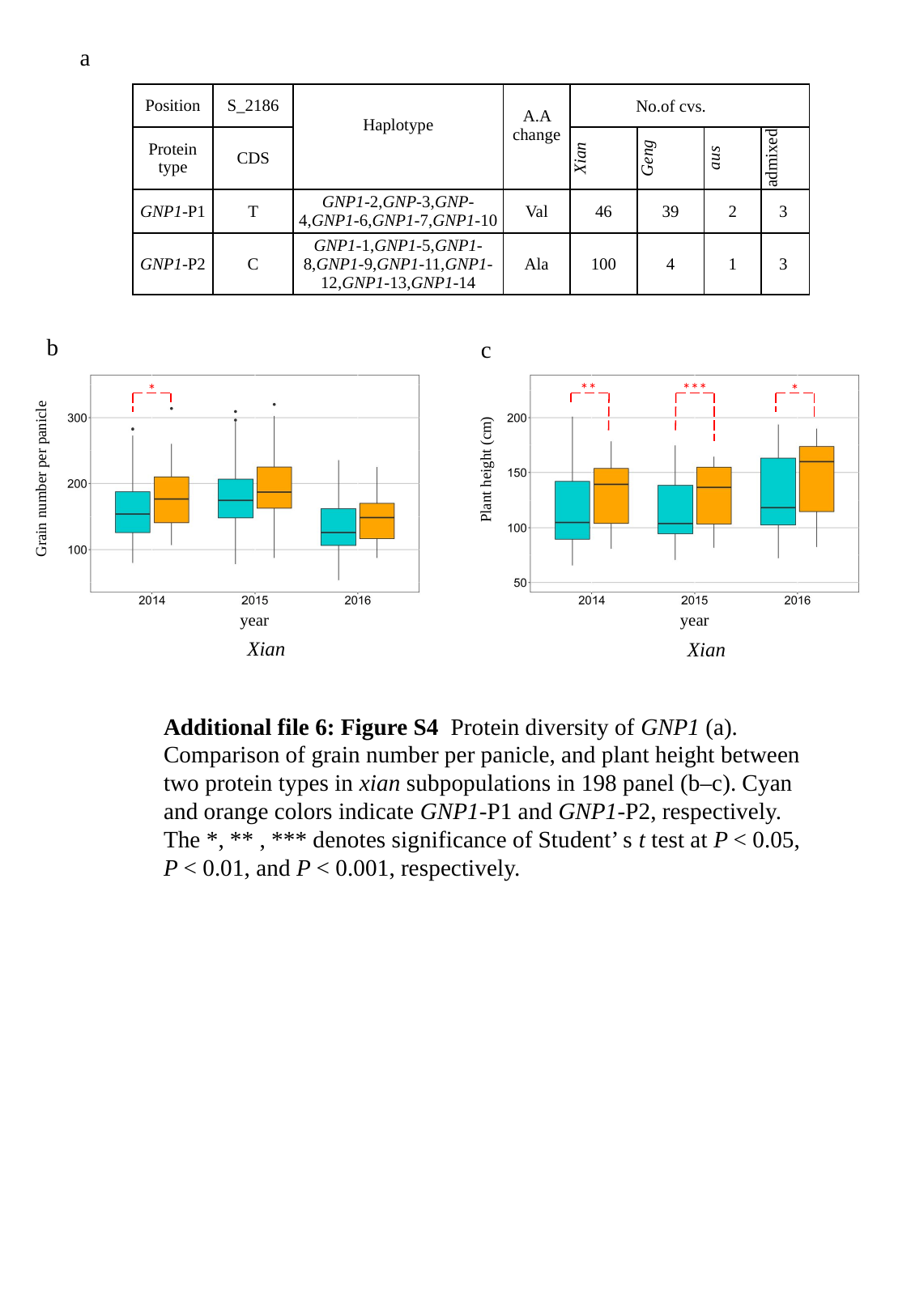

a
| Position | S\_2186 | Haplotype | A.A change | No.of cvs. | | | |
| --- | --- | --- | --- | --- | --- | --- | --- |
| Protein type | CDS | | | Xian | Geng | aus | admixed |
| GNP1-P1 | T | GNP1-2,GNP-3,GNP-4,GNP1-6,GNP1-7,GNP1-10 | Val | 46 | 39 | 2 | 3 |
| GNP1-P2 | C | GNP1-1,GNP1-5,GNP1-8,GNP1-9,GNP1-11,GNP1-12,GNP1-13,GNP1-14 | Ala | 100 | 4 | 1 | 3 |
b
c
***
**
*
*
Plant height (cm)
Grain number per panicle
year
year
Xian
Xian
Additional file 6: Figure S4 Protein diversity of GNP1 (a). Comparison of grain number per panicle, and plant height between two protein types in xian subpopulations in 198 panel (b–c). Cyan and orange colors indicate GNP1-P1 and GNP1-P2, respectively. The *, ** , *** denotes significance of Student’ s t test at P < 0.05, P < 0.01, and P < 0.001, respectively.
