# Supplementary material for: Natural Sequence Variations and Combinations of GNP1 and NAL1 Determine the Grain Number per Panicle in Rice
Source: Rice (N Y). 2020 Feb 28;13:14. doi: 10.1186/s12284-020-00374-8 (PMC7048901; doi:10.1186/s12284-020-00374-8)
Supplement: Supplementary file 7 — Additional file 7 : Figure S5. Expression level of NAL1 and GNP1 haplotypes in young panicle at the panicle initiation stage. (a) Expression level of GNP1–1 and GNP1–6 in xian subpopulation. (b) Expression level of NAL1–2, NAL1–4 and NAL1–6 in geng subpopulation. (c) Expression level of NAL1–1, NAL1–2 and NAL1–3 in xian subpopulation. Error bars indicate SD; Letters are ranked by Duncan’s test at P < 0.05. The * denotes significance of Student’ s t test at P < 0.05. [file 12284_2020_374_MOESM7_ESM.ppt]

## Slide 1
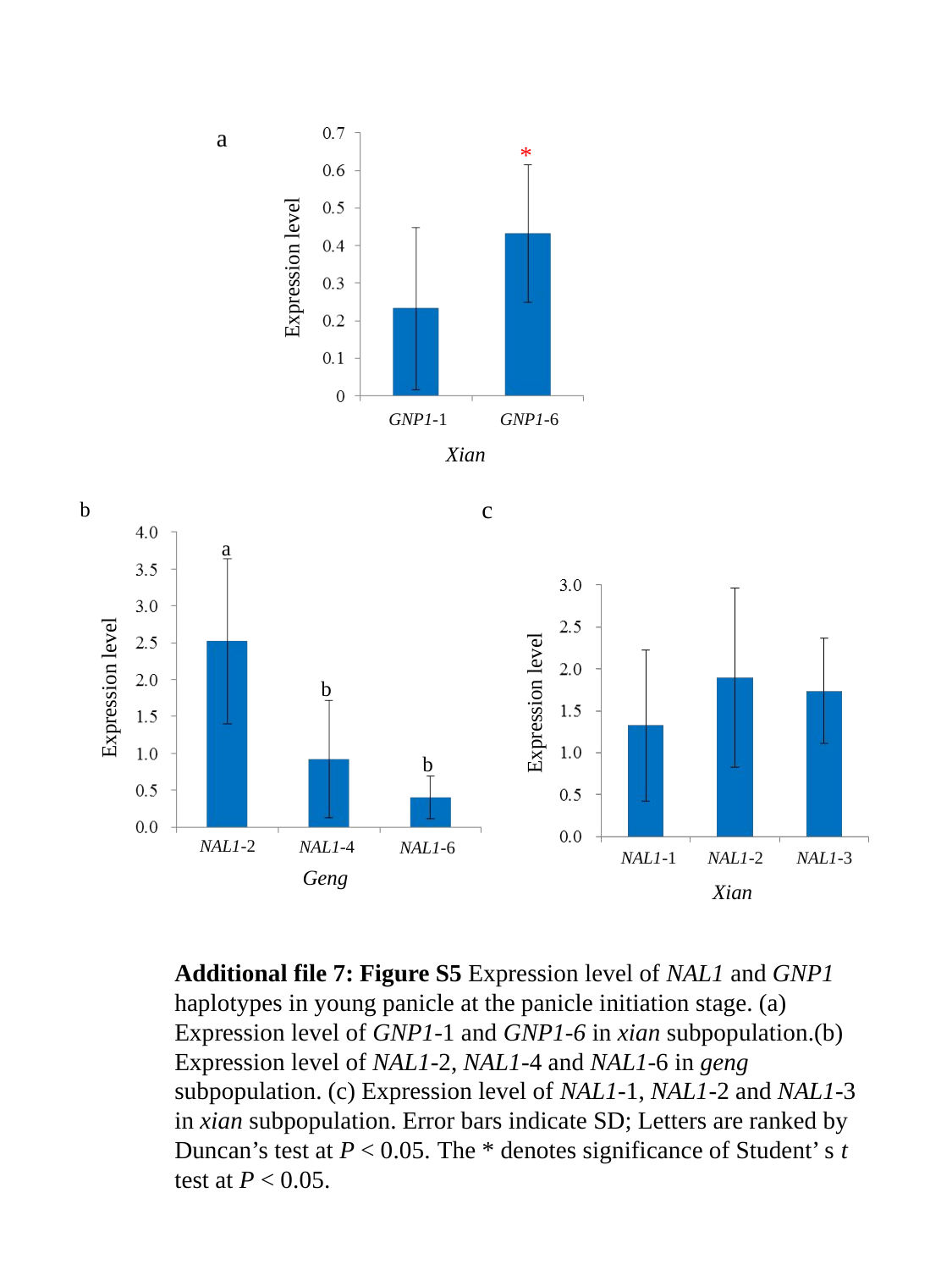

a
*
Expression level
GNP1-1
GNP1-6
Xian
c
b
a
Expression level
NAL1-1
NAL1-2
NAL1-3
Xian
Expression level
b
b
NAL1-2
NAL1-4
NAL1-6
Geng
Additional file 7: Figure S5 Expression level of NAL1 and GNP1 haplotypes in young panicle at the panicle initiation stage. (a) Expression level of GNP1-1 and GNP1-6 in xian subpopulation.(b) Expression level of NAL1-2, NAL1-4 and NAL1-6 in geng subpopulation. (c) Expression level of NAL1-1, NAL1-2 and NAL1-3 in xian subpopulation. Error bars indicate SD; Letters are ranked by Duncan’s test at P < 0.05. The * denotes significance of Student’ s t test at P < 0.05.
