# Supplementary material for: Natural Sequence Variations and Combinations of GNP1 and NAL1 Determine the Grain Number per Panicle in Rice
Source: Rice (N Y). 2020 Feb 28;13:14. doi: 10.1186/s12284-020-00374-8 (PMC7048901; doi:10.1186/s12284-020-00374-8)
Supplement: Supplementary file 8 — Additional file 8 : Figure S6. Protein diversity of NAL1 (a). Comparison of grain number per panicle, and flag leaf width among three protein types in xian and geng subpopulations, respectively (b–e). Cyan, orange, and lightpink colors indicate NAL1-P1, NAL1-P2 and NAL1-P3, respectively. The *, **, *** denotes significance of Student’ s t test at P < 0.05, P < 0.01, and P < 0.001, respectively. [file 12284_2020_374_MOESM8_ESM.pptx]

## Slide 1
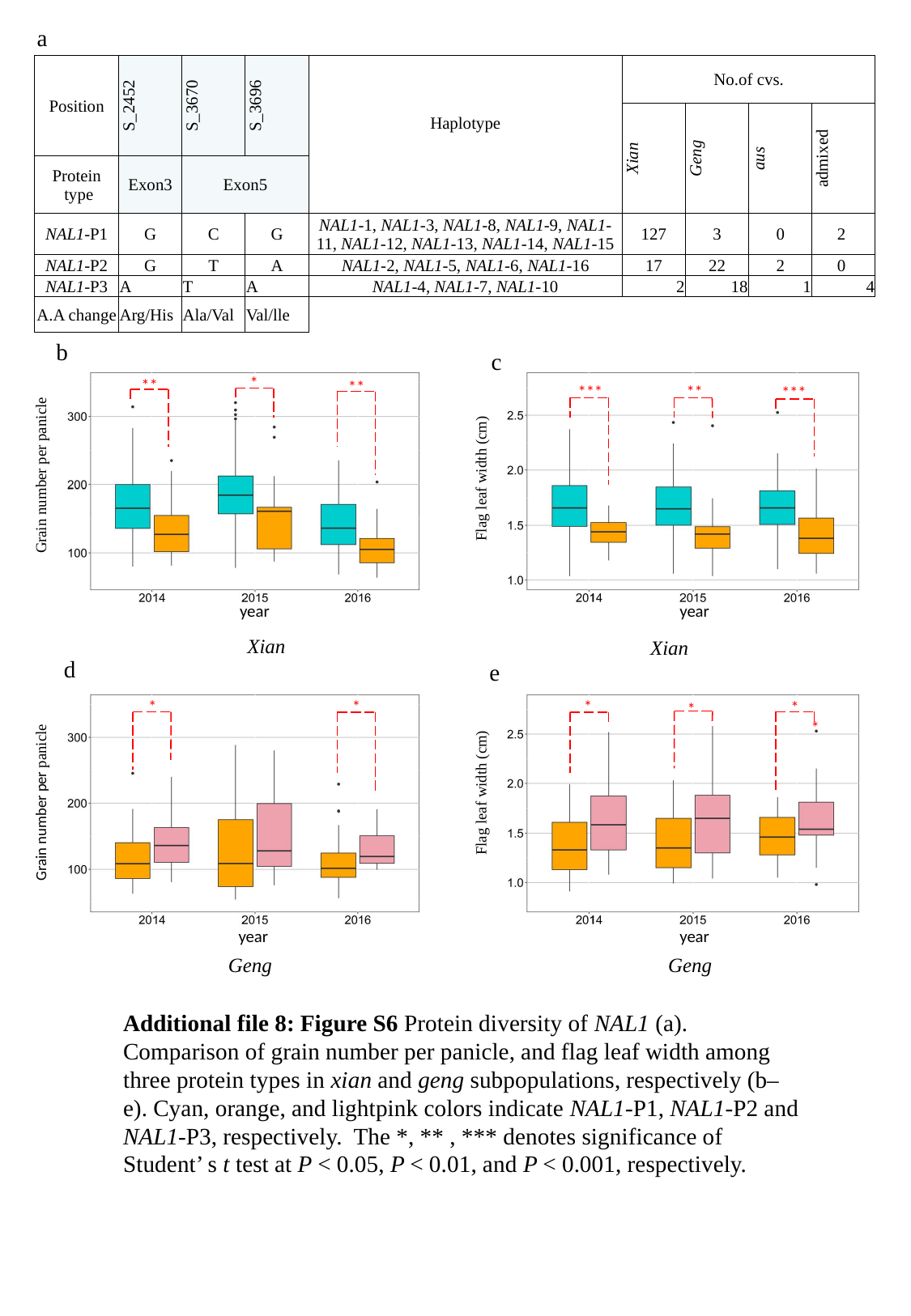

a
| Position | S\_2452 | S\_3670 | S\_3696 | Haplotype | No.of cvs. | | | |
| --- | --- | --- | --- | --- | --- | --- | --- | --- |
| | | | | | Xian | Geng | aus | admixed |
| Protein type | Exon3 | Exon5 | | | | | | |
| NAL1-P1 | G | C | G | NAL1-1, NAL1-3, NAL1-8, NAL1-9, NAL1-11, NAL1-12, NAL1-13, NAL1-14, NAL1-15 | 127 | 3 | 0 | 2 |
| NAL1-P2 | G | T | A | NAL1-2, NAL1-5, NAL1-6, NAL1-16 | 17 | 22 | 2 | 0 |
| NAL1-P3 | A | T | A | NAL1-4, NAL1-7, NAL1-10 | 2 | 18 | 1 | 4 |
| A.A change | Arg/His | Ala/Val | Val/lle | | | | | |
b
c
*
**
**
***
**
***
Grain number per panicle
Flag leaf width (cm)
year
year
Xian
Xian
d
e
*
*
*
*
*
Flag leaf width (cm)
Grain number per panicle
*
year
year
Geng
Geng
Additional file 8: Figure S6 Protein diversity of NAL1 (a).
Comparison of grain number per panicle, and flag leaf width among three protein types in xian and geng subpopulations, respectively (b–e). Cyan, orange, and lightpink colors indicate NAL1-P1, NAL1-P2 and NAL1-P3, respectively. The *, ** , *** denotes significance of Student’ s t test at P < 0.05, P < 0.01, and P < 0.001, respectively.
