# Supplementary material for: Natural Sequence Variations and Combinations of GNP1 and NAL1 Determine the Grain Number per Panicle in Rice
Source: Rice (N Y). 2020 Feb 28;13:14. doi: 10.1186/s12284-020-00374-8 (PMC7048901; doi:10.1186/s12284-020-00374-8)
Supplement: Supplementary file 9 — Additional file 9 : Table S3. Variance components and heritabilty estimated by multiple-site analysis. [file 12284_2020_374_MOESM9_ESM.docx]

**Additional file 9: Table S3** Variance components and heritabilty estimated by multiple-site analysis

| GB | Trait | *V*_GEI_ | *V*_G_ | Rep(Env) | *V*_E_ | *V*_e_ | *h*^2^ |
| --- | --- | --- | --- | --- | --- | --- | --- |
| TQ-ILs | PN | 2.7 | 0.8 | 0.0 | 7.4 | 3.8 | 0.3 |
|  | TGW | 0.7 | 2.6 | 0.0 | 0.0 | 1.2 | 0.8 |
|  | GNP | 275.0 | 705.7 | 37.0 | 493.9 | 396.6 | 0.7 |
|  | GY | 41.7 | 49.5 | 0.2 | 172.5 | 53.4 | 0.6 |
| LT-ILs | PN | 1.6 | 1.0 | 0.9 | 3.9 | 4.2 | 0.4 |
|  | TGW | 0.9 | 2.7 | 0.0 | 0.9 | 0.9 | 0.8 |
|  | GNP | 96.6 | 639.5 | 37.8 | 294.2 | 1029.0 | 0.7 |
|  | GY | 60.5 | 14.9 | 10.8 | 221.0 | 92.1 | 0.2 |

GB, genetic background; PN, panicle number per plant; TGW, thousand-grain weight; GNP, grain number per panicle; GY, grain yield per plant; *V*_E_, environmental variance; *V*_G_, genotypic variance; *V*_GEI_, genotype-by-environment interaction variance; *V*_e_, residual variance; Rep (Env), replication variance within environment; *h*^2^, narrow-sense heritability.
