# Supplementary material for: Natural Sequence Variations and Combinations of GNP1 and NAL1 Determine the Grain Number per Panicle in Rice
Source: Rice (N Y). 2020 Feb 28;13:14. doi: 10.1186/s12284-020-00374-8 (PMC7048901; doi:10.1186/s12284-020-00374-8)
Supplement: Supplementary file 10 — Additional file 10 : Figure S7. Comparison of grain number per panicle, flag leaf width and effective panicle number between R-type and H-type alleles located in the third exon of NAL1 gene in 3 K panel in xian and geng subpopulation, respectively. ***, P < 0.001 (Student’ s t test). [file 12284_2020_374_MOESM10_ESM.ppt]

## Slide 1
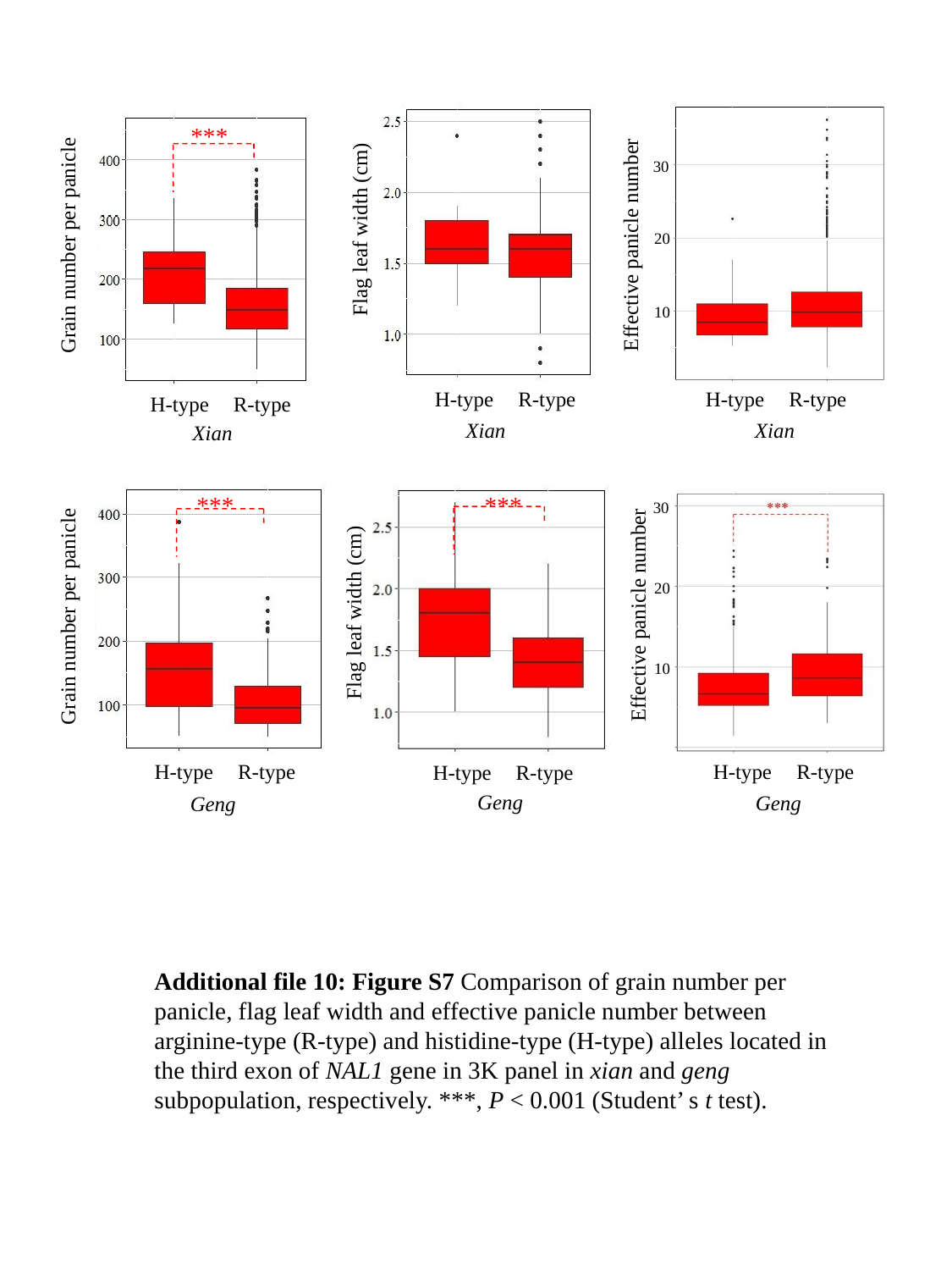

***
Grain number per panicle
H-type
R-type
Xian
30
Flag leaf width (cm)
Effective panicle number
20
10
H-type
R-type
H-type
R-type
Xian
Xian
***
Grain number per panicle
H-type
R-type
Geng
***
30
Flag leaf width (cm)
20
Effective panicle number
10
H-type
R-type
H-type
R-type
Geng
Geng
Additional file 10: Figure S7 Comparison of grain number per panicle, flag leaf width and effective panicle number between arginine-type (R-type) and histidine-type (H-type) alleles located in the third exon of NAL1 gene in 3K panel in xian and geng subpopulation, respectively. ***, P < 0.001 (Student’ s t test).
