# Supplementary material for: Natural Sequence Variations and Combinations of GNP1 and NAL1 Determine the Grain Number per Panicle in Rice
Source: Rice (N Y). 2020 Feb 28;13:14. doi: 10.1186/s12284-020-00374-8 (PMC7048901; doi:10.1186/s12284-020-00374-8)
Supplement: Supplementary file 11 — Additional file 11 : Table S4. Distribution of protein mutation sites diversity at amino acid position 233 of NAL1 gene. R, arginine; H, histidine. [file 12284_2020_374_MOESM11_ESM.docx]

**Additional file 11: Table S4** Distribution of protein mutation sites diversity at amino acid position 233 of *NAL1* gene

| Population | Group | N | R-type allele | H-type allele | |
| --- | --- | --- | --- | --- | --- |
|  | *O. sativa* | 198 | 173 | 25 | |
| 198 accessions | ssp. *Geng* | 43 | 25 (58.1%) | 18(41.9%) | |
|  | ssp. *Xian* | 146 | 144 (98.6%) | 2 (1.4%) | |
|  | *O. rufipogon* | 8 | 8 | 0 | |
|  | *O. sativa* | 2628 | 1963 | 638 |  |
| 3K RGP | ssp. *Geng* | 839 | 211 (25.1%) | 616 (73.4%) |  |
|  | ssp. *Xian* | 1785 | 1752 (98.2%) | 18 (1.0%) |  |

R, arginine; H, histidine
