# Supplementary material for: Natural Sequence Variations and Combinations of GNP1 and NAL1 Determine the Grain Number per Panicle in Rice
Source: Rice (N Y). 2020 Feb 28;13:14. doi: 10.1186/s12284-020-00374-8 (PMC7048901; doi:10.1186/s12284-020-00374-8)
Supplement: Supplementary file 12 — Additional file 12 : Figure S8. Geographic distributions of different haplotypes of NAL1 and GNP1 among the 28 areas sampled. [file 12284_2020_374_MOESM12_ESM.pdf]

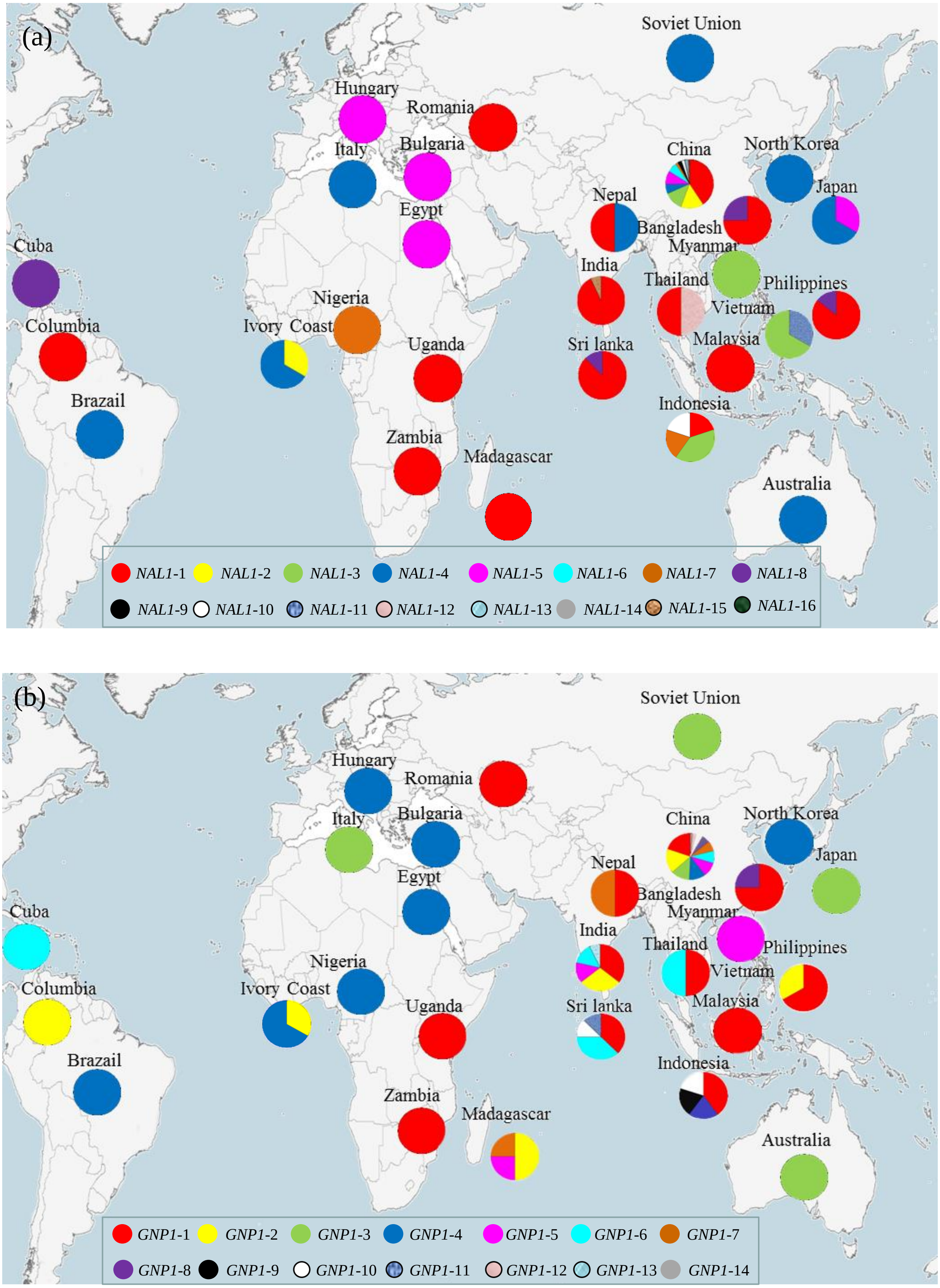

Additional file 12:Figure S8 Geographic distributions of different haplotypes of *NAL1* (a) and *GNP1* (b) among the 28 areas sampled. Each circle is constructed with the respective frequencies of these haplotypes at each locality.
