# Supplementary material for: Natural Sequence Variations and Combinations of GNP1 and NAL1 Determine the Grain Number per Panicle in Rice
Source: Rice (N Y). 2020 Feb 28;13:14. doi: 10.1186/s12284-020-00374-8 (PMC7048901; doi:10.1186/s12284-020-00374-8)
Supplement: Supplementary file 14 — Additional file 14 : Table S6. Sequencing primers used in this study. [file 12284_2020_374_MOESM14_ESM.docx]

**Additional file 14:Table S6** Sequencing primers used in this study

| Primer name | sequence (5′ - 3′) | Restriction Enzyme Set  PCR Product vs (Plasmid) |
| --- | --- | --- |
| 1. sequence | | |
| *GNP1*-SQ-pf | GCTGTTGCGATGGTTGAGACGA |  |
| *GNP1*-SQ-pr | CCATATGAACTGCGACGGGATCTC |  |
| *GNP1*-SQ-cf | GTGGCACCATCCCTTTCA |  |
| *GNP1*-SQ-cr | CCCCACCCAAGGTTTAGC |  |
| *NAL1*-SQ-pf | AGTGTGAACTGCTCTCTAGCCTAGATC |  |
| *NAL1*-SQ-pr | CTGCACAATGCTGTAGGTTGGATGTC |  |
| *NAL1*-SQ-gf | ATGAAGCCTTCGGACGATAAGGCG |  |
| *NAL1*-SQ-gr | TCATTTCTCCAGGTCAAGGCTTGATCC |  |
| *NAL1*-SQ-uf | GGTTGATCTTGGCCGTCTACTC |  |
| *NAL1*-SQ-ur | CTTACGCCTCTTCCTCTGTGTC |  |
| 1. real-time PCR | | |
| *GNP1*-RT-f | CACCTTCATGGCGCTCTC |  |
| *GNP1*-RT-r | CCACCGTGTCCATCTCCG |  |
| *NAL1*-RT-f | GCCTTATTATCCTGACTAGCCAA |  |
| *NAL1*-RT-r | ATCAACCCCACTAGTCCA |  |
| 3.complementary test | | |
| *GNP1*-CT-f | ACGCGGGATCCGATCGCCAATATCAACAAGCAGCTCA | *BamH*I/*EcoR*I(*BamH*I/*EcoR*I) |
| *GNP1*-CT-r | ACGCGGAATTCAGGCGTCCTCCTTCTTTCCTTGTC |  |
| *NAL1-*CT-pf | ACGCGGGATCCTTGTTCGTGACCGTTGGCTTGAG | *BamH*I/*Not*I(*BamH*I/*Sbf*I) |
| *NAL1-*CT-pr | GAGCCATCCCCACAACCGTC |  |
| *NAL1-*CT-cf | CGACGACCTCTCCCGCTACATG | *Not*I/*Sbf*I(*BamH*I/*Sbf*I) |
| *NAL1-*CT-cr | ACGCGCCTGCAGGCATCAAACTTGGACCAGAGATGTGCT |  |
| *GNP1*-CTP-f (*GNP1*+*NAL1*) | ACGCGCCTGCAGGGATCGCCAATATCAACAAGCAGCTCA | *Sbf*I/*Pst*I(*Sbf*I/*Pst*I) |
| *GNP1*-CTP-r (*GNP1*+*NAL1*) | ACGCGCTGCAGAGGCGTCCTCCTTCTTTCCTTGTC |  |
